# Supplementary material for: Carbene-stabilized enantiopure heterometallic clusters featuring EQE of 20.8% in circularly-polarized OLED
Source: Nat Commun. 2023 Jul 11;14:4121. doi: 10.1038/s41467-023-39802-w (PMC10336039; doi:10.1038/s41467-023-39802-w)
Supplement: Supplementary file 1 — Supplementary Information [file 41467_2023_39802_MOESM1_ESM.pdf]

Supplementary Information

Carbene-Stabilized Enantiopure Heterometallic Clusters Featuring EQE of 20.8% in Circularly-Polarized OLED

Xiao-Hong Ma[1†], Jing Li[1†], Peng Luo[2], Jia-Hua Hu[1], Zhen Han[1], Xi-Yan Dong[1,2✉], Guohua Xie[3✉] & Shuang-Quan Zang[1✉]

[1]Henan Key Laboratory of Crystalline Molecular Functional Materials, Henan International Joint Laboratory of Tumor Theranostical Cluster Materials, Green Catalysis Center, and College of Chemistry, Zhengzhou University, Zhengzhou 450001, China.

[2]College of Chemistry and Chemical Engineering Henan Polytechnic University Jiaozuo 454000, China.

[3]Sauvage Center for Molecular Sciences, Hubei Key Lab on Organic and Polymeric Optoelectronic Materials, Department of Chemistry, Wuhan University, Wuhan 430072, China

✉Corresponding authors E-mail: zangsqzg@zzu.edu.cn, dongxiyan0720@hpu.edu.cn, guohua.xie@whu.edu.cn

Table of Contents

Supplementary Methods

1. Materials and reagents

2. General characterization

3. Photophysical measurements

4. Synthesis and characterizations

4.1. Synthesis of *R/S*-NHC^{py}-H ·PF_6

4.2. Synthesis of *R/S*-NHC^{ql}-H ·PF_6

4.3 Synthesis of *R/S*-[Au(NHC^{py})$_2$]PF_6 and *R/S*-[Au(NHC^{ql})$_2$]PF_6.

4.4. Synthesis of *R/S*-ql-X (X = Cl, Br, and I), *R/S*-py-X (X = Br and I)

5. Crystallographic data collection and refinement of the structure.

6. Quantum chemical calculations

7. Boltzmann Equation

8. Transient absorption (TA) measurement.

9. Device fabrication

Supplementary Discussion

10. Supplementary figures and tables

10.1. Characterization of ligands and precursors

10.2. Single crystal structure of clusters

10.3. Single crystal data of clusters

10.4. Characterization of clusters

10.5. Luminescence spectra of clusters

10.6. Theoretical calculations

10.7. CD and CPL spectra

10.8. OLED

Supplementary References

Supplementary Methods

1. Materials and reagents.

Ammonium hexafluorophosphate, (1*S*, 2*S*)-(-)-1, 2-Diphenyl-1, 2-ethanediamine, (1*R*, 2*R*)-(+)-1, 2-Diphenyl-1,2-ethanediamine, glyoxylic acid monohydrate, NBS, $Na_2S_2O_5$, NaOH, 2-(Chloromethyl)quinoline hydrochloride and 2-Picolyl chloride hydrochloride were purchased from Energy. Me_2SAuCl was prepared according to the literature method[1]. *R*/*S*-NHC^{ql}-H·PF_6 and *R*/*S*-NHC^{py}-H·PF_6 were prepared according to modification of the literature method[2-3]. Other reagents and solvents for synthesis were obtained from commercial sources and used without further purification.

2. General characterization.

Powder X-ray diffraction (PXRD) patterns of the samples were recorded on a D/MAX-3D diffractometer. Variable-temperature PXRD patterns were recorded on a Rigaku SmartLabSE diffractometer with Cu-Kα radiation. ^{1}H NMR spectra were performed on a Bruker DRX spectrometer operating at 600 MHz in $CDCl_3$ or CD_2Cl_2. Fourier transform infrared (FT-IR) spectra were analyzed on a Nexus 870 FT-IR spectrometer from KBr pellets as the sample matrix. Elemental analyses (EA) were carried out with a Perkin-Elmer 240 elemental analyzer. HRESI-TOF-MS spectra were collected on an AB Sciex X500R Q-TOF spectrometer. Thermogravimetric analyses (TGA) were performed on a TA Q50 system from room temperature (RT) to 800 °C at a heating rate of 10 °C/min under a nitrogen atmosphere.

3. Photophysical measurements.

UV-vis absorption spectra were recorded using a Hitachi U-2000 UV-visible spectrophotometer. Emission and excitation spectra at RT were recorded with a HORIBA FluoroLog-3 fluorescence spectrometer. Variable-temperature steady-state emission spectra of solid-state *R*-py-X (X = Br and I) and *R*-ql-X (X = Cl, Br, and I) were measured on a HORIBA FluoroLog-3 fluorescence spectrometer, which was recorded at each temperature after five minutes maintenance. The photoluminescence quantum efficiency was measured using an integrating sphere on a HORIBA Scientific Fluorolog-3 spectrofluorometer. Luminescence decay was measured on a HORIBA

Fluorolog-3 fluorescence spectrometer equipped with a 355 nm laser operating in time-correlated single-photon counting mode (TCSPC). Luminescence microscopy images were recorded on an Olympus BX53 microscope. The Circular dichroism (CD), Circularly polarized luminescence (CPL) spectra and Circularly polarized electroluminescence (CPEL) were recorded on a Chirascan V100 spectropolarimeter and a JASCO CPL-300 spectrometer, respectively.

4. Synthesis and characterizations.

4.1. Synthesis of *R*-NHC^{py}-H·PF_6.

(a) The mixture of (1*R*, 2*R*)-(+)-1,2-Diphenyl-1,2-ethanediamine (3.00 g, 14.11 mmol) and glyoxylic acid monohydrate (1.50 g, 16.50 mmol) in MeOH (100 mL) was stirred at room temperature for 4 h. NBS (3.30 g, 18.00 mmol) was added to the mixture and the resulting solution was stirred overnight at room temperature. The reaction was quenched by the addition of sat. $Na_2S_2O_5$ aq., and MeOH was evaporated in vacuo. 5 % NaOH aq. was added to the residue, and the solution was extracted with AcOEt. The organic layer was dried over $MgSO_4$, and evaporated in vacuo. The residue was purified by SiO_2 column chromatography to give product. Yield: 60%.

(b) 2-(Chloromethyl)pyridine hydrochloride (3.35 g, 20.40 mmol), (*R*, *R*)-4,5-diphenyl-imidazoline (2.22 g, 10 mmol) and $NaHCO_3$ (2.52 g, 31.12 mmol) were taken up in ethanol (100 mL) and refluxed for 2 days. The solvent was removed in vacuo, the residue taken up in DCM and dried over $MgSO_4$, and the solution filtered. Removal of the DCM in vacuo gave an oil that was triturated with 15 ml THF to give a powder. This was further washed with THF and dried in vacuo. Yield: (75%).

(c) In a flask, *R*-NHC^{py}-H·Cl (0.54 g, 1 mmol) was dissolved in 3 mL methanol, and NH_4PF_6 (0.48 g. 3 mmol) aqueous solution was added dropwise. The precipitate was formed during 2 h. The reaction mixture was filtered, washed thoroughly with water/methanol, and dried in vacuo. Yield: (90%). The synthesis was the same as that of *R*-NHC^{py}-PF_6 ligand except for the use of (1*S*, 2*S*)-(-)-1,2-Diphenyl-1,2-ethanediamine in place of (1*R*, 2*R*)-(+)-1,2-Diphenyl-1,2-ethanediamine. ^{1}H NMR (600 MHz, $CDCl_3$): δ 11.08 (s, 1H), δ 8.60 (d, 2H), δ 7.72 (t, 2H), δ 7.45 (d, 2H), δ 7.37-7.42 (m, 6H), δ 7.27-7.31 (m, 6H), δ 5.52 (d, 2H), δ 5.16 (s, 2H), δ 4.30 (d, 2H). ESI-

MS (m/z): calcd. for $C_{27}H_{25}N_4$ [M^+], 405.2206; found, 405.2202.

4.2. Synthesis of *R/S*-NHC^{ql}-$H{\cdot}PF_6$.

The synthesis was the same as that of *R/S*-NHC^{ql}-$H{\cdot}PF_6$ ligand except for the use of 2-(chloromethyl)quinoline hydrochloride in place of 2-picolyl chloride hydrochloride. ^1H NMR (600 MHz, $CDCl_3$): δ 11.21 (s, 1H), δ 8.12 (d, 2H), δ 8.04 (d, 2H), δ 7.79 (d, 2H), δ 7.73 (t, 2H), δ 7.53 (t, 2H), δ 7.34-7.41 (m, 20H), δ 5.76 (d, 2H), δ 5.38 (s, 2H), δ 4.47 (d, 2H). ESI-MS (m/z): calcd. for $C_{35}H_{29}N_4$ [M^+], 505.2313; found, 505.2364.

4.3. Synthesis of *R/S*-$[Au(NHC^{py})_2]PF_6$ and *R/S*-$[Au(NHC^{ql})_2]PF_6$.

R/S-NHC^{ql}-$H{\cdot}PF_6$ (0.65 g, 1 mmol) or *R/S*-NHC^{py}-$H{\cdot}PF_6$ (0.54 g, 1 mmol), Ag_2O (66 mg, 0.28 mmol), and about 40 mg of $^nBu_4PF_6$ in 40 mL of CH_2Cl_2 was added. The mixture was protected from light and stirred for 10 min at room temperature. NaOH (1 M, 3 mL) was then added, and stirring was continued for 4 h. The mixture was filtered through Celite, and the clear filtrate was reduced to the minimum volume under vacuum. *R/S*-$[Ag(NHC^{ql})_2]PF_6$ or *R/S*-$[Ag(NHC^{py})_2]PF_6$ was precipitated as a white powder by the addition of diethyl ether. Next, a 25 mL round-bottom flask was charged with *R/S*-$[Ag(NHC^{ql})_2]PF_6$ (0.38 mg, 0.3 mmol) or *R/S*-$[Ag(NHC^{py})_2]PF_6$ (0.32 mg, 0.3 mmol) in 30 mL of CH_2Cl_2. Me_2SAuCl (0.096 g, 0.3 mmol) in 10 mL of CH_2Cl_2 was added dropwise. The mixture was protected from light and stirred for 30 min during which time a precipitate formed. The solution was filtered through Celite removing the precipitated AgCl. The clear filtrate was subsequently reduced to 2 mL, and a white powder was precipitated with diethyl ether. Yield: *R*-$[Au(NHC^{py})_2]PF_6$, 72%; *S*-$[Au(NHC^{py})_2]PF_6$, 75%; *S*-$[Au(NHC^{py})_2]PF_6$, 78% and *R/S*-$[Au(NHC^{ql})_2]PF_6$, 76% (based on *R/S*-NHC^{py}-$H{\cdot}PF_6$ and *R/S*-NHC^{ql}-$H{\cdot}PF_6$).

4.4. Synthesis of *R/S*-ql-X (X = Cl, Br, and I), *R/S*-py-X (X = Br and I).

R/S-$[Au(NHC^{ql})_2]PF_6$ or *R/S*-$[Au(NHC^{py})_2]PF_6$ (0.05 mmol) was dissolved in 6 mL of CH_2Cl_2. To this was added CuX (0.2 mmol) (X = Cl, Br, and I) suspended in 6 mL of CH_2Cl_2. The solution was stirred for an additional 3 h during which a yellow solution formed. The mixture was filtered through Celite, and the clear filtrate was reduced to 2 mL, then a yellow powder was precipitated with diethyl ether. The yellow powder was collected by filtration yielding. The yellow powder was recrystallized from CH_2Cl_2 and

diethyl ether to produce yellow crystals. For *R*-py-Br, ^{1}H NMR (600 MHz, CD_2Cl_2): δ 8.83 (d, 4H), δ 7.27-7.40 (m, 8H), δ 7.06-7.19 (m, 12H), δ 6.94-7.05 (m, 8H), δ 6.49 (d, 4H), δ 5.67 (d, 4H), δ 4.93 (s, 4H), δ 4.32 (d, 4H). Elemental analysis (calculated): C, 38.16%; H, 3.23%; and N, 6.09%; found: C, 37.86%; H, 3.19%; and N, 6.14%. For *S*-py-Br, ^{1}H NMR (600 MHz, CD_2Cl_2): δ 8.72 (d, 4H), δ 7.16-7.25 (m, 8H), δ 6.96-7.06 (m, 12H), δ 6.86-6.93 (m, 8H), δ 6.38 (d, 4H), δ 5.56 (d, 4H), δ 4.82 (s, 4H), δ 4.21 (d, 4H). Elemental analysis (calculated): C, 38.16%; H, 3.23%; and N, 6.09%; found: C, 37.83%; H, 3.25%; and N, 6.06%. For *R*-py-I, ^{1}H NMR (600 MHz, CD_2Cl_2): δ 8.86 (t, 4H), δ 7.32 (t, 8H), δ 7.07-7.23 (m, 12H), δ 6.90-7.04 (m, 8H), δ 6.46 (t, 4H), δ 5.67 (d, 4H), δ 4.90 (s, 4H), δ 4.40 (d, 4H). Elemental analysis (calculated): C, 35.06%; H, 2.94%; and N, 5.64%; found: C, 34.93%; H, 2.82%; and N, 5.24%. For *S*-py-I, ^{1}H NMR (600 MHz, CD_2Cl_2): δ 8.75 (t, 4H), δ 7.21 (t, 8H), δ 6.95-7.14 (m, 12H), δ 6.81-6.94 (m, 8H), δ 6.36 (t, 4H), δ 5.55 (d, 4H), δ 4.76 (s, 4H), δ 4.29 (d, 4H). Elemental analysis (calculated): C, 35.06%; H, 2.94%; and N, 5.64%; found: C, 35.07%; H, 2.84%; and N, 5.46%. For *R*-ql-Cl, ^{1}H NMR (600 MHz, CD_2Cl_2): δ 9.20 (d, 4H), δ 8.22 (s, 3H), δ 8.07 (s, 3H), δ 7.91 (s, 3H), δ 7.73 (s, 5H), δ 7.05-7.30 (m, 16H), δ 6.41-7.02 (m, 10H), δ 6.00 (d, 4H),δ 4.87 (s, 1H), δ 4.55 (d, 4H), δ 4.22 (s, 3H). Elemental analysis (calculated): C, 48.47%; H, 3.45%; and N, 6.28%; found: C, 48.00%; H, 3.45%; and N, 6.17%. For *S*-ql-Cl, ^{1}H NMR (600 MHz, CD_2Cl_2): δ 9.20 (d, 4H), δ 8.21 (s, 3H), δ 8.07 (s, 3H), δ 7.92 (s, 3H), δ 7.73 (s, 5H), δ 7.03-7.26 (m, 16H), δ 6.37-7.01 (m, 10H), δ 5.96 (d, 4H),δ 4.87 (s, 1H), δ 4.53 (d, 4H), δ 4.22 (s, 3H). Elemental analysis (calculated): C, 48.47%; H, 3.45%; and N, 6.28%; found: C, 48.02%; H, 3.37%; and N, 6.22%. For *R*-ql-Br, ^{1}H NMR (600 MHz, CD_2Cl_2): δ 9.22 (d, 4H), δ 7.63-8.29 (m, 17H), δ 6.87-7.25 (m, 13H), δ 6.72 (m, 4H), δ 6.42-6.60 (m, 6H), δ 6.02 (d, 4H),δ 4.83 (s, 2.4H), δ 4.54 (d, 4H), δ 4.17 (s, 1.6H). Elemental analysis (calculated): C, 43.60%; H, 3.47%; and N, 5.28%; found: C, 43.40%; H, 3.39%; and N, 5.40%. For *S*-ql-Br, ^{1}H NMR (600 MHz, CD_2Cl_2): δ 9.22 (d, 4H), δ 7.62-8.27 (m, 17H), δ 6.87-7.25 (m, 13H), δ 6.72 (m, 4H), δ 6.42-6.59 (m, 6H), δ 6.02 (d, 4H),δ 4.82 (s, 2.4H), δ 4.56 (d, 4H), δ 4.17 (s, 1.6H). Elemental analysis (calculated): C, 43.60%; H, 3.47%; and N, 5.28%; found: C, 43.38%; H, 3.42%; and N, 5.26%. For *R*-ql-I, ^{1}H NMR (600 MHz, CD_2Cl_2):

δ 9.22 (d, 4H), δ 7.62-8.22 (m, 17H), δ 6.81-7.28 (m, 10H), δ 6.24-6.79 (m, 13H), δ 6.10 (d, 4H), δ 4.78 (s, 3.4H), δ 4.65 (d, 4H), δ 4.15 (s, 0.6H). Elemental analysis (calculated): C, 40.64%; H, 3.04%; and N, 5.12%; found: C, 40.60%; H, 3.02%; and N, 5.06%. For *S*-ql-I, ^{1}H NMR (600 MHz, CD_2Cl_2): δ 9.34 (d, 4H), δ 7.70-8.36 (m, 17H), δ 6.92-7.43 (m, 10H), δ 6.38-6.89 (m, 13H), δ 6.22 (d, 4H), δ 4.90 (s, 3.5H), δ 4.76 (d, 4H), δ 4.27 (s, 0.5H). Elemental analysis (calculated): C, 40.64%; H, 3.04%; and N, 5.12%; found: C, 40.64%; H, 3.14%; and N, 5.01%.

5. Crystallographic data collection and refinement of the structure. *R/S*-py-X (X = Br and I) and *R/S*-ql-X (X = Cl, Br, and I) were measured by single-crystal X-ray diffraction (SCXRD) with a Bruker diffractometer at 200 K, using Mo-Kα radiation (λ = 0.71073 Å). *R*-ql-I was also measured at different temperatures (100, 150, 180, 200, 250, and 300 K). The intensities were corrected for absorption using the empirical method implemented in SCALE3 ABSPACK scaling algorithm. The structures were solved with intrinsic phasing methods (SHELXT-2015), and refined by full-matrix least-squares on F^2 using *OLEX2*[4], which utilizes the SHELXL-2015 module. The least-squares refinement of the structural model was performed under hard geometry restraints and displacement parameter restraints due to the weak diffraction and serious disorder of PF_6^-, Et_2O and CH_2Cl_2 molecules in the lattice, such as ISOR, SADI, SIMU, and DFIX. Solvent molecules of all clusters have been identified and further confirmed by thermogravimetric and elemental analysis. All host molecular atoms were refined anisotropically, and the hydrogen atoms were included in idealized positions. The crystallographic data were listed in Supplementary Tables 1-6 and 11.

For *R*-py-Br, due to the weak diffraction and serious disorder of PF_6^- and CH_2Cl_2 molecules in the lattice, we used SADI restraints C-Cl bond of CH_2Cl_2 molecular; used ISOR restraints F and C atoms of PF_6^- and CH_2Cl_2 molecular. The twin law was tested but refined to BASF of zero, and hence removed. Two outlier reflections were omitted from the refinements.

For *S*-py-Br, due to the weak diffraction and disorder of PF_6^- and CH_2Cl_2 molecules in the lattice, we used ISOR restraints F and C atoms of PF_6^- and CH_2Cl_2 molecular.

For *R*-py-I, due to the weak diffraction and serious disorder of PF_6^- and Et_2O molecules in the lattice, we used SADI and DFIX restraints P-F bond of PF_6^- molecular; we used DFIX restraints C-C and C-O bonds of Et_2O molecular; we used ISOR restraints F atoms of PF_6^-. Two outlier reflections were omitted from the refinements.

For *S*-py-I, due to the weak diffraction and serious disorder of Et_2O molecules in the lattice, we used DFIX restraints C-C and C-O bonds of Et_2O molecular; we used SIMU restraints O and C atoms of Et_2O molecular. One outlier reflection was omitted from the refinements.

For *R*-ql-Cl, due to the weak diffraction and serious disorder of Et_2O molecules in the lattice, we used DFIX restraints C-C and C-O bond of Et_2O molecular; we used SIMU and ISOR restraints F and C atoms of PF_6^- and Et_2O. The twin law was tested but refined to BASF of zero, and hence removed.

For *S*-ql-Cl, due to the weak diffraction and serious disorder of $PF_6^-$and Et_2O molecules in the lattice, we used DFIX restraints C-C and C-O bond of Et_2O molecular; we used SIMU and ISOR restraints F and C atoms of $PF_6^-$and Et_2O; we used ISOR restraints disorder C atoms of NHC^{ql} ligand. The twin law was tested but refined to BASF of zero, and hence removed.

For *R*-ql-Br, due to the weak diffraction and serious disorder of Et_2O molecules in the lattice, we used DFIX restraints C-C and C-O bonds of Et_2O molecular; used ISOR and SIMU restraints O and C atoms of Et_2O molecular; used ISOR restraints disorder C atoms of NHC^{ql} ligand. The twin law was tested but refined to BASF of zero, and hence removed. Four outlier reflections were omitted from the refinements.

For *S*-ql-Br, due to the weak diffraction and serious disorder of Et_2O molecules in the lattice, we used DFIX restraints C-C and C-O bonds of Et_2O molecular; used ISOR restraints disorder C atoms of NHC^{ql} ligand; used EDAP and EXYZ restraints C atom of Et_2O and CH_2Cl_2 to share one atomic coordinate. Seven outlier reflections were omitted from the refinements.

For *R*-ql-I-100 K, due to the weak diffraction and serious disorder of Et_2O molecules in the lattice, we used DFIX restraints C-C and C-O bonds of Et_2O molecular; we used SIMU restraints O and C atoms of Et_2O molecular. The twin law was tested but refined

to BASF of zero, and hence removed. Two outlier reflections were omitted from the refinements.

For *R*-ql-I-150 K, due to the weak diffraction and serious disorder of Et_2O molecules in the lattice, we used DFIX restraints C-C and C-O bonds of Et_2O molecular. One outlier reflection was omitted from the refinements.

For *R*-ql-I-180 K, due to the weak diffraction and serious disorder of Et_2O molecules in the lattice, we used DFIX restraints C-C and C-O bonds of Et_2O molecular; we used SIMU restraints O and C atoms of Et_2O molecular. Five outlier reflections were omitted from the refinements.

For *R*-ql-I-200 K, due to the weak diffraction and serious disorder of Et_2O molecules in the lattice, we used DFIX restraints C-C and C-O bonds of Et_2O molecular; we used SIMU restraints O and C atoms of Et_2O molecular; we used ISOR restraints disorder C atoms of NHC^{ql} ligand. Eight outlier reflections were omitted from the refinements.

For *S*-ql-I-200 K, due to the weak diffraction and serious disorder of Et_2O molecules in the lattice, we used DFIX restraints C-C and C-O bonds of Et_2O molecular; we used SIMU restraints O and C atoms of Et_2O molecular; we used ISOR restraints disorder C atoms of NHC^{ql} ligand. The twin law was tested but refined to BASF of zero, and hence removed. Two outlier reflections were omitted from the refinements.

For *R*-ql-I-250 K, due to the weak diffraction and serious disorder of Et_2O molecules in the lattice, we used DFIX restraints C-C and C-O bonds of Et_2O molecular; we used SIMU restraints O and C atoms of Et_2O molecular. We used ISOR restraints disorder C atoms of NHC^{ql} ligand. Three outlier reflections were omitted from the refinements.

For *R*-ql-I-300 K, due to the weak diffraction and serious disorder of Et_2O molecules in the lattice, we used DFIX restraints C-C and C-O bonds of Et_2O molecular; used SIMU restraints O and C atoms of Et_2O molecular. We used ISOR restraints disorder C atoms of NHC^{ql} ligand. The twin law was tested but refined to BASF of zero, and hence removed. Three outlier reflections were omitted from the refinements.

6. Quantum chemical calculations.

In this study, the PL origin of a series of ligand-protected Au(I)-Cu(I) alloy clusters (*R*-py-X (X = Br and I) and *R*-ql-X (X = Cl, Br, and I)) has been studied by the density

functional theory (DFT) and time-dependent density functional theory (TD-DFT) calculations. The DFT and TD-DFT calculations were carried out using the Gaussian 16 program[5]. The hybrid PBE0 functional in conjunction with def2-SVP basis set were used for geometric optimization of the ground state and excited state configuration of ligand-protected Au(I)-Cu(I) alloy clusters. Well-converged geometries are obtained by tightening the energy and gradient convergence criteria to 1×10^{-6} Hartree and 1×10^{-3} Hartree/Å, respectively. The theoretical UV-Vis spectra of *R*-py-X (X = Br and I) and *R*-ql-X (X = Cl, Br, and I) were calculated at the optimized ground-state (S_0) geometries using TD-DFT under PBE0/def2SVP level. SMD solvent model (dichloromethane) was applied for TD-DFT calculations. The calculated absorption spectra were obtained from Multiwfn 3.8[6]. The excited state gradients were calculated to optimize the excited-state geometry. For *R*-ql-X (X = Cl and Br) the first singlet excited state (S_1) and the low-lying triplet excited states (T_n, $n = 1, 2$) were optimized. For *R*-ql-I and *R*-py-I, during the structure optimization of T_2, we found that the energies of T_2 and T_1 are almost degenerate, and the energy gap between S_1 and T_1 geometries is very small, the states primarily relevant for the ISC are the S_1 and T_1 states.

The hole and electron pair distribution analyses were performed using the Multiwfn code[6-7]. The input wave function for the analysis of hole and electron pair distribution of ligand-protected Au(I)-Cu(I) alloy clusters were calculated using the Gaussian 16 program[5]. The *Sr* index is defined as the full space integration of a function (S_r(r)) describing the overlap between electron and hole distributions, formulated as $S_r(\mathrm{r})\ index = \int \sqrt{\rho^{hole}(r)\rho^{electron}(r)}dr$, where $\rho^{hole}(r)$ and $\rho^{electron}(r)$ are the hole and electron distribution. *D* index is the distance between a hole and an electron center of mass, formulated as $D\ \text{index} = \sqrt{(D_x)^2 + (D_y)^2 + (D_z)^2}$.

The Kohn−Sham (KS) orbital energy levels were calculated based on the PBE0 functional and the all-electron TZP basis set using the Amsterdam density functional (ADF 2016) software packages[8]. Scalar relativistic effects are included by utilizing the zeroth-order regular approximation (ZORA).

The radiative rate of fluorescence (k_f) and phosphorescence (k_p) were evaluated by

Einstein spontaneous emission relationship:

$$k_r = \frac{8\pi^2 v_{fi}^3}{3\varepsilon_0 \hbar c^3}\mu_{fi}^2 \approx \frac{f v_{fi}^2}{1.499} \qquad (1)$$

where f is the dimensionless oscillator strength, μ is the transition dipole moment, and v is the transition energy in wavenumber.

For the non-radiative transition processes, the present study focuses on the most important step of TADF, namely the $S_1 \rightarrow T_n$ (n=1, 2) intersystem crossing (ISC) process and $T_n \rightarrow S_1$ (n=1, 2) reverse intersystem crossing (RISC) process. The ISC rate constant (k_{ISC}) and RISC rate constant (k_{RISC}) between the lowest singlet excited state (S_1) and a triplet excited state (T_n) are obtained by the semiclassical Marcus theory expression (the equation (2)):[9-11]

$$k_{ISC(RISC)} = \frac{2\pi}{\hbar} V_{SOC}^2 \frac{1}{\sqrt{4\pi\lambda\kappa_B T}} \exp\left[-\frac{(\Delta E_{ST} + \lambda)^2}{4\lambda\kappa_B T}\right] \qquad (2)$$

where the ΔE_{ST} is the adiabatic energy difference between the S_1 and T_n states. The k_B is the Boltzmann constant, and the temperature (T) is taken to be 298.15K. V_{SOC} is the spin-orbit coupling matrix elements (SOCME) were calculated by ORCA 5.0.0 software package[12] based on the PBE0 functional and the DKH-def2-TZVP(-f) basis set (SARC-DKH-TZVP for Au and I atoms). For the $S_1 \rightarrow T_n$ ISC process, the SOCME is calculated based on the optimized S_1 geometry. For the $T_n \rightarrow S_1$ RISC process, the SOCME is calculated based on the optimized T_n geometry. The contributions of the three degenerate triplet states ($T_{n,x}$, $T_{n,y}$, and $T_{n,z}$) were taken into account by calculating the root sum square of the real and imaginary parts (Re and Im) of the matrix elements, as expressed by the following equation:

$$|V_{soc}|^2 = \frac{|\langle S_1|\hat{H}soc|T_n\rangle|^2}{3} = \frac{1}{3}\sum_{J=x,y,z} \langle S_1|\hat{H}soc|T_n{}^J\rangle\langle S_1|\hat{H}soc|T_n{}^J\rangle^* \qquad (3)$$

It is noted that the reorganization energy can be represented as a sum of the contributions from the surroundings and those from intramolecular vibrations. Generally, the contribution of reorganization energy from the surroundings to the system is small, and the calculation error is large, which can be ignored. Therefore, it is generally believed that the reorganization energy of the system mainly comes from

the contribution of the intramolecular vibrations, and only the internal reorganization energy is calculated. At present, the most common and simple and effective method for calculating the reorganization energy is to use the classical method. For the $S_1 \rightarrow T_n$ ISC process, the reorganization energy is the energy gap between T_n energy level at the optimized S_1 geometries and T_n energy level at the optimized T_n geometries. For the $T_n \rightarrow S_1$ process, the reorganization energy is the energy gap between S_1 energy level at the optimized T_n geometries and S_1 energy level at the optimized S_1 geometries.

The interna conversion rate constant (k_{IC}) between the T_n and T_{n-1} are obtained by the semiclassical Marcus theory expression (the equation (4)):[13-15]

$$k_{T_nT_{n-1}} = \frac{2\pi}{\hbar} V_{SOC}^2 \frac{1}{\sqrt{4\pi\lambda\kappa_B T}} \exp\left[-\frac{\left(\Delta E_{T_nT_{n-1}} + \lambda\right)^2}{4\lambda\kappa_B T}\right] \quad (4)$$

where the $\Delta E_{T_nT_{n-1}}$ is the adiabatic energy difference between the T_n and T_{n-1} states. The k_B is the Boltzmann constant, and the temperature (T) is taken to be 298.15K. V_{SOC} is the spin-orbit coupling matrix elements (SOCME). For the $T_n \rightarrow T_{n-1}$ IC process, the SOCME is calculated based on the optimized T_n geometry. The contributions of the three degenerate triplet states ($T_{n,x}$, $T_{n,y}$, $T_{n,z}$ and $T_{n-1,x}$, $T_{n-1,y}$, $T_{n-1,z}$) were taken into account by calculating the root sum square of the real and imaginary parts (Re and Im) of the matrix elements, as expressed by the following equation:

$$|V_{soc}|^2 = \frac{\left|\langle T_n|\hat{H}soc|T_{n-1}\rangle\right|^2}{3}$$

$$= \frac{1}{3} \sum_{J=x,y,z} \langle T_n{}^J|\hat{H}soc|T_{n-1}{}^J\rangle \langle T_n{}^J|\hat{H}soc|T_{n-1}{}^J\rangle^* \quad (5)$$

For calculating the reorganization energy is to use the classical method, which is the energy gap between T_n energy level at the optimized T_{n-1} geometries and T_n energy level at the optimized T_n geometries.

The minimum energy crossing point (MECP) structure have calculated by TD-DFT using sobMECP Program (Lu, T. (2020) sobMECP Program. http://sobereva.com/286 (Accessed Nov 6, 2020)) and Gaussian 16 program. The MECP is recognized when the energy gap is smaller than 0.0001 Hartree and the gradient criteria are met. The hybrid PBE0 functional in conjunction with Def2-SVP basis set are used for MECP geometric

optimization of *R*-ql-Cl, *R*-ql-Br, *R*-ql-I between S_1 and T_1/T_2. At the MECP structures, the SOCME were calculated by ORCA 5.0.0 software package based on the PBE0 functional and the DKH-def2-TZVP(-f) basis set (SARC-DKH-TZVP for Au and I atoms).

The basis function contribution to the hole and electron of the MECP structures and the geometries of S_1 and T_1/T_2 were calculated. The hole and electron pair distribution analyses were performed using the Multiwfn code. The input wave function for the analysis of hole and electron pair distribution was calculated using the Gaussian 16 program.

7. Boltzmann Equation.

$$\tau = \frac{3 \ + \ \exp\left[-\frac{\Delta E(\mathrm{S_1} - \mathrm{T_1})}{k_B T}\right]}{3k(\mathrm{T_1}) \ + \ k(\mathrm{S_1})\exp\left[-\frac{\Delta E(\mathrm{S_1} - \mathrm{T_1})}{k_B T}\right]}$$

where k_B is Boltzmann constant, τ is the experimental average lifetime, $\Delta E(S_1\text{-}T_1)$ is the energy gap between S_1 and T_1 states, $k(T_1) = 1/\tau(T_1)$ and $k(S_1) = 1/\tau(S_1)$ are the decay rates with decay times $\tau(T_1)$ and $\tau(S_1)$ of the triplet and singlet excited states, respectively, and T is the temperature.

8. Transient absorption (TA) measurement.

The TA spectra were recorded on a commercial pump-probe system (Helios, Ultrafast Systems LLC) in combination with a femtosecond laser system (Astrella, Coherent). Laser pulses (~800 nm center wavelength, < 100 fs duration, 1 kHz repetition rate, ~7 mJ/pulse) were generated by a Ti:sapphire-based regenerative amplifier laser system. The laser pulses were split to generate pump and probe beams. The pump pulses at 360 nm were delivered by an optical parametric amplifier (OPA) that was excited by a portion of the 800-nm laser pulses. The pump pulse energy in each measurement was ~25 μJ/cm^2 at the sample cell. The probe and reference beams were generated by focusing the 800-nm beam (split from the amplifier with a tiny portion) onto a CaF_2 crystal to generate white-light continuum pulses (370-650 nm). The pump-probe delay was controlled by an optical delay line.

9. Device fabrication

The OLEDs were fabricated with the structure of [ITO/PEDOT:PSS (50 nm)/9,9'-(1,3-Phenylene)bis-9H-carbazole (mCP):***S*-ql-I** (40 nm)/Bis[2-(diphenylphosphino)phenyl] ether oxide (DPEPO) (10 nm)/1,3,5-tri(3-pyridyl)-phen-3-ylbenzene (TmPyPB) (50 nm)/8-hydroxyquinolatolithium (Liq) (1 nm)/Al (100 nm)]. The pre-patterned ITO glass was consecutively cleaned with ultrasonication in deionized water, acetone, and ethanol, followed by N_2 drying and UV-O_3 treatment for 20 min prior to use. The hole injection layer PEDOT:PSS was spin-coated onto the ITO substrates at 4000 rpm and subsequently annealed at 120 °C for 10 min and then the samples were transferred to the N_2-filled glovebox. The emitting layer mCP/***S*-ql-I** (98:2, wt./wt.%)) was spin-coated onto PEDOT:PSS directly from a chlorobenzene solution at a concentration of 10 mg/mL. All the samples were annealed at 50 °C for 10 min before being loaded into the vacuum chamber. The hole-blocking layer DPEOP and the electron transporting layer TmPyPB layers were thermally deposited onto the emitting layer to enhance the charge balance in the emitting layer. Finally, the composite Liq/Al cathode was evaporated. All the devices were encapsulated with UV-curable resin before taken out the glove-box. The current-voltage-luminance characteristics and the EL spectra were measured at ambient air simultaneously with customized software controlling a PR735 spectrascan spectrometer and a Keithley 2400 source measurement unit.

Supplementary Discussion

10. Supplementary figures and tables

10.1. Characterization of ligands and precursors

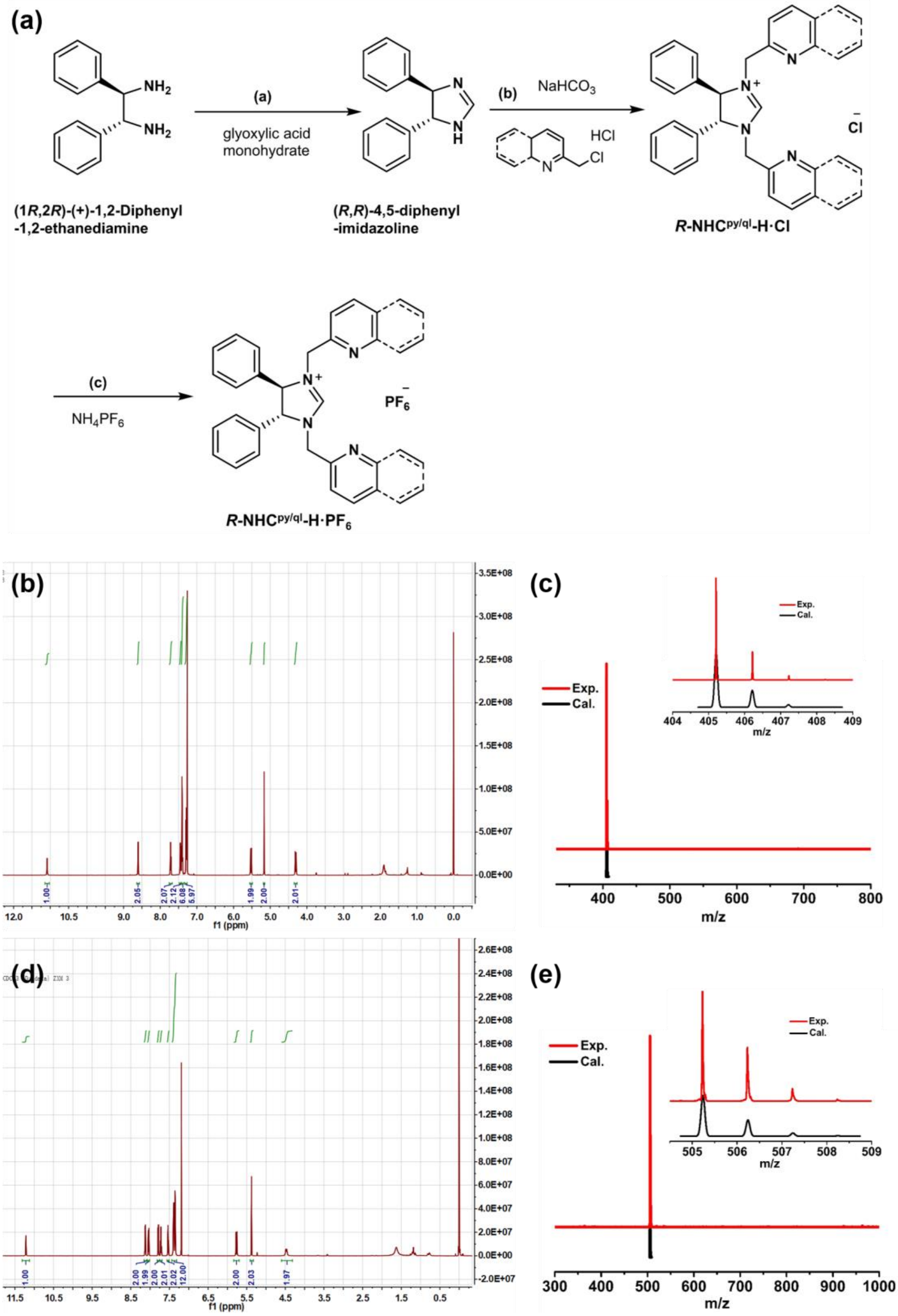

Supplementary Figure 1. (a) Synthesis of *R/S*-NHC$^{py/ql}$-H·PF$_6$ ligands. ^{1}H NMR spectrum of (b) *R*-NHC^{py}-H·PF$_6$ and (d) *R*-NHC^{ql}-H PF$_6$ (CDCl$_3$). Positive mode ESI-MS of (c) *R*-NHC^{py}-H·PF$_6$ and (e) *R*-NHC^{ql}-H PF$_6$ (CH$_2$Cl$_2$). Insets: Enlarged portion of the ESI-MS exhibiting the measured and simulated isotopic distribution patterns.

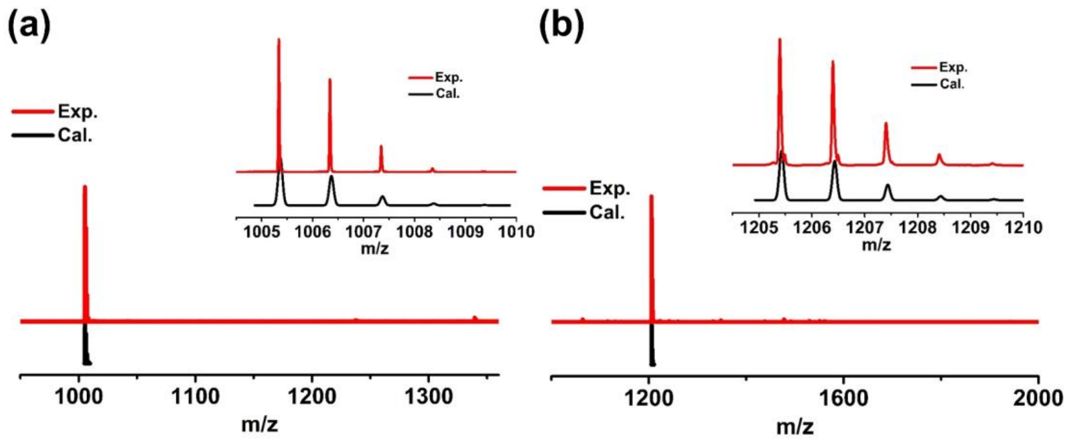

Supplementary Figure 2. (a) Positive mode ESI-MS of *R*-[Au(NHC^{py})$_2$]PF_6 dissolved in dichloromethane (m/z = 1005.3745). Insets: Enlarged portion of the ESI-MS exhibiting the measured (red line) and simulated (black line) isotopic distribution patterns in the m/z range of 900-1400 with a charge state of +1. (b) Positive mode ESI-MS of *R*-[Au(NHC^{ql})$_2$]PF_6 dissolved in dichloromethane (m/z = 1205.4320). Insets: Enlarged portion of the ESI-MS exhibiting the measured (red line) and simulated (black line) isotopic distribution patterns in the m/z range of 1000-2000 with a charge state of +1.

10.2. Single crystal structure of clusters

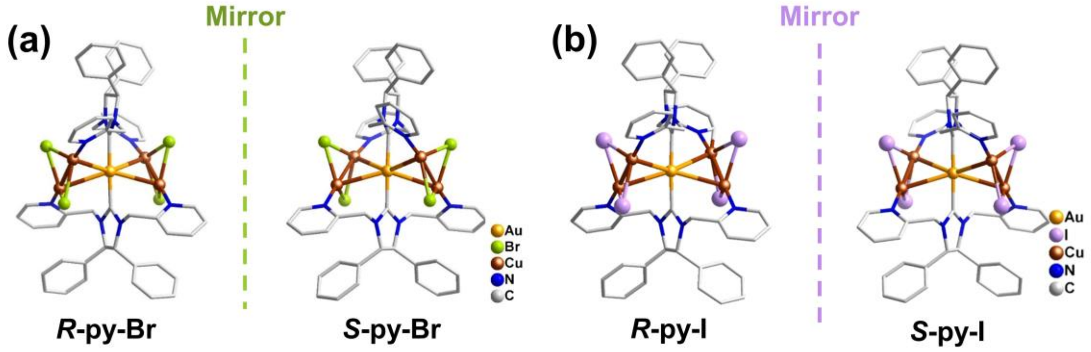

Supplementary Figure 3. Structure of the enantiomers of (a) *R/S*-py-Br (b) *R/S*-py-I.

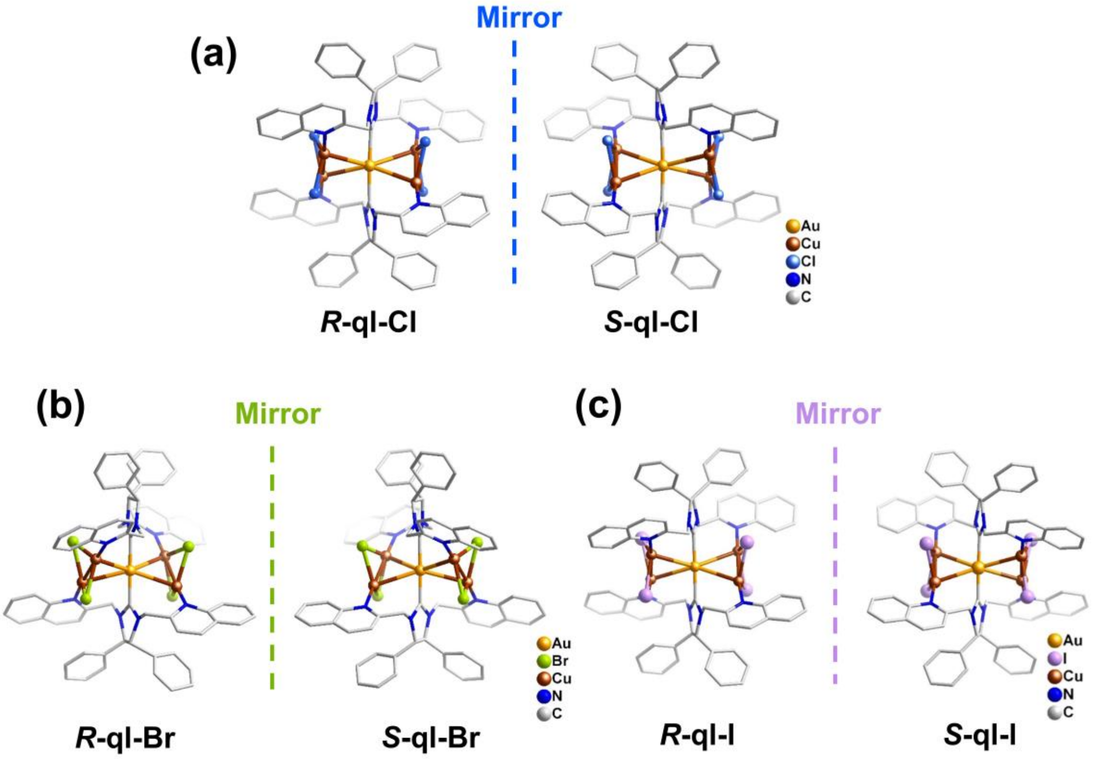

Supplementary Figure 4. Structure of the enantiomers of (a) *R/S*-ql-Cl, (b) *R/S*-ql-Br (c) *R/S*-ql-I.

10.3. Single crystal data of clusters

Supplementary Table 1. Crystal data and structure refinement for *R*/*S*-ql-I

Compound	*R*-ql-I	*S*-ql-I
CCDC number	2225247	2225253
Empirical formula	$C_{74}H_{66}AuCu_4F_6I_4N_8OP$	$C_{74}H_{66}AuCu_4F_6I_4N_8OP$
Formula weight	2187.04	2187.04
Temperature/K	200	200
Crystal system	orthorhombic	orthorhombic
Space group	$P2_12_12$	$P2_12_12$
a/Å	26.7253(11)	26.701(2)
b/Å	19.5313(7)	19.506(2)
c/Å	14.9188(7)	14.9367(14)
α/°	90	90
β/°	90	90
γ/°	90	90
Volume/Å^3	7787.3(6)	7779.6(13)
Z	4	4
ρ_{calc} g/cm^3	1.865	1.867
μ/mm^{-1}	4.62	4.624
F(000)	4200	4200
Crystal size/mm^3	0.12 × 0.04 × 0.04	0.15 × 0.05 × 0.05
Radiation	MoKα (λ = 0.71073)	MoKα (λ = 0.71073)
2θ range for data collection/°	3.758 to 54.986	3.758 to 55.03
Index ranges	$-34 \le h \le 34$, $-25 \le k \le 25$, $-19 \le l \le 19$	$-34 \le h \le 34$, $-25 \le k \le 25$, $-19 \le l \le 19$
Reflections collected	487450	506974
Independent reflections	17859 [R_{int} = 0.0935, R_{sigma} = 0.0255]	17875 [R_{int} = 0.1018, R_{sigma} = 0.0279]
Data/restraints/parameters	17859/25/889	17875/23/889
Goodness-of-fit on F^2	1.036	1.078
Final R indexes [I>=2σ (I)]	R_1 = 0.0250, wR_2 = 0.0596	R_1 = 0.0265, wR_2 = 0.0699
Final R indexes [all data]	R_1 = 0.0317, wR_2 = 0.064	R_1 = 0.0317, wR_2 = 0.0751
Largest diff. peak/hole / e Å^{-3}	0.75/-1.06	1.67/-1.87
Flack parameters	0.0017(18)	0.004(4)

$R_1=\Sigma||F_o| - |F_c||/\Sigma|F_o|$, $wR_2=[\Sigma[w(F_o^2 - F_c^2)^2]/\Sigma w(F_o^2)^2]^{1/2}$.

Supplementary Table 2. Crystal data and structure refinement for *R/S*-py-Br

Compound	*R*-py-Br	*S*-py-Br
CCDC number	2225239	2225240
Empirical formula	$C_{58.5}H_{59}AuBr_4ClCu_4F_6N_8OP$	$C_{58.5}H_{59}AuBr_4ClCu_4F_6N_8OP$
Formula weight	1841.32	1841.32
Temperature/K	200	200
Crystal system	orthorhombic	orthorhombic
Space group	$P2_12_12_1$	$P2_12_12_1$
a/Å	15.2743(7)	15.2964(9)
b/Å	15.7324(8)	15.7426(10)
c/Å	27.9349(14)	27.9235(18)
α/°	90	90
β/°	90	90
γ/°	90	90
Volume/Å^3	6712.8(6)	6724.1(7)
Z	4	4
ρ_{calc} g/cm^3	1.822	1.819
μ/mm^{-1}	5.93	5.92
F(000)	3580	3580
Crystal size/mm^3	0.12 × 0.12 × 0.03	0.12 × 0.11 × 0.03
Radiation	MoKα (λ = 0.71073)	MoKα (λ = 0.71073)
2θ range for data collection/°	3.9 to 55.03	3.712 to 55.098
Index ranges	$-19 \leq h \leq 19$, $-20 \leq k \leq 20$, $-36 \leq l \leq 36$	$-19 \leq h \leq 19$, $-20 \leq k \leq 20$, $-36 \leq l \leq 36$
Reflections collected	381690	375047
Independent reflections	15439 [R_{int} = 0.0738, R_{sigma} = 0.0250]	15484 [R_{int} = 0.1076, R_{sigma} = 0.0333]
Data/restraints/parameters	15439/55/828	15484/72/835
Goodness-of-fit on F^2	1.163	1.161
Final R indexes [I>=2σ (I)]	R_1 = 0.0309, wR_2 = 0.0810	R_1 = 0.0386, wR_2 = 0.0911
Final R indexes [all data]	R_1 = 0.0357, wR_2 = 0.0865	R_1 = 0.0509, wR_2 = 0.1006
Largest diff. peak/hole / e Å^{-3}	1.22/-1.11	1.43/-2.05
Flack parameters	0.008(7)	0.015(8)

$R_1=\Sigma||F_o| - |F_c||/\Sigma|F_o|$, $wR_2=[\Sigma[w(F_o^2 - F_c^2)^2]/\Sigma w(F_o^2)^2]^{1/2}$.

Supplementary Table 3. Crystal data and structure refinement for *R/S*-py-I

Compound	*R*-py-I	*S*-py-I
CCDC number	2225252	2225251
Empirical formula	$C_{58}H_{58}AuCu_4F_6I_4N_8P$	$C_{58}H_{58}AuCu_4F_6I_4N_8P$
Formula weight	1986.82	1986.82
Temperature/K	200	200
Crystal system	tetragonal	tetragonal
Space group	$I4_1$	$I4_1$
a/Å	17.6372(5)	17.6980(10)
b/Å	17.6372(5)	17.6980(10)
c/Å	22.5259(7)	22.5113(15)
α/°	90	90
β/°	90	90
γ/°	90	90
Volume/Å^3	7007.2(5)	7051.0(9)
Z	4	4
ρ_{calc} g/cm^3	1.883	1.872
μ/mm^{-1}	5.123	5.092
F(000)	3784	3784
Crystal size/mm^3	0.14 × 0.12 × 0.1	0.12 × 0.1 × 0.1
Radiation	MoKα (λ = 0.71073)	MoKα (λ = 0.71073)
2θ range for data collection/°	4.618 to 55.006	4.604 to 54.968
Index ranges	$-22 \leq h \leq 22$, $-22 \leq k \leq 22$, $-29 \leq l \leq 29$	$-22 \leq h \leq 22$, $-22 \leq k \leq 22$, $-29 \leq l \leq 29$
Reflections collected	199395	209824
Independent reflections	8039 [R_{int} = 0.0518, R_{sigma} = 0.0184]	8072 [R_{int} = 0.0504, R_{sigma} = 0.0169]
Data/restraints/parameters	8039/24/394	8072/9/374
Goodness-of-fit on F^2	1.138	1.150
Final R indexes [I>=2σ (I)]	R_1 = 0.0228, wR_2 = 0.0672	R_1 = 0.0174, wR_2 = 0.0484
Final R indexes [all data]	R_1 = 0.0274, wR_2 = 0.0740	R_1 = 0.0211, wR_2 = 0.0557
Largest diff. peak/hole / e Å^{-3}	0.81/-1.26	0.83/-0.85
Flack parameters	0.0012(18)	0.0039(16)

$R_1=\Sigma||F_o| - |F_c||/\Sigma|F_o|$, $wR_2=[\Sigma[w(F_o^2 - F_c^2)^2]/\Sigma w(F_o^2)^2]^{1/2}$.

Supplementary Table 4. Crystal data and structure refinement for *R/S*-ql-Cl

Compound	*R*-ql-Cl	*S*-ql-Cl
CCDC number	2225238	2225237
Empirical formula	$C_{72}H_{61}AuCl_6Cu_4F_6N_8O_{0.5}P$	$C_{72}H_{61}AuCl_6Cu_4F_6N_8O_{0.5}P$
Formula weight	1784.18	1784.18
Temperature/K	200	200.00(10)
Crystal system	orthorhombic	orthorhombic
Space group	$C222_1$	$C222_1$
a/Å	15.2368(11)	15.2263(15)
b/Å	25.7783(11)	25.7708(15)
c/Å	18.9604(11)	18.9483(15)
α/°	90	90
β/°	90	90
γ/°	90	90
Volume/Å^3	7447.2(8)	7435.2(10)
Z	4	4
ρ_{calc} g/cm^3	1.591	1.594
μ/mm^{-1}	3.313	3.319
F(000)	3540	3540
Crystal size/mm^3	0.12 × 0.03 × 0.03	0.13 × 0.05 × 0.03
Radiation	MoKα (λ = 0.71073)	MoKα (λ = 0.71073)
2θ range for data collection/°	3.776 to 55.108	3.778 to 55.832
Index ranges	$-19 \leq h \leq 19, -33 \leq k \leq 33, -24 \leq l \leq 24$	$-19 \leq h \leq 19, -33 \leq k \leq 33, -24 \leq l \leq 24$
Reflections collected	248898	211371
Independent reflections	8593 [R_{int} = 0.0999, R_{sigma} = 0.0286]	8748 [R_{int} = 0.1158, R_{sigma} = 0.0384]
Data/restraints/parameters	8593/50/455	8748/40/442
Goodness-of-fit on F^2	1.034	1.030
Final R indexes [I>=2σ (I)]	R_1 = 0.0285, wR_2 = 0.0718	R_1 = 0.0307, wR_2 = 0.0735
Final R indexes [all data]	R_1 = 0.0359, wR_2 = 0.0755	R_1 = 0.0461, wR_2 = 0.0792
Largest diff. peak/hole / e Å^{-3}	0.66/-0.68	0.64/-0.77
Flack parameters	0.009(3)	0.008(3)

$R_1=\Sigma||F_o| - |F_c||/\Sigma|F_o|$, $wR_2=[\Sigma[w(F_o^2 - F_c^2)^2]/\Sigma w(F_o^2)^2]^{1/2}$.

Supplementary Table 5. Crystal data and structure refinement for *R/S*-ql-Br

Compound	*R*-ql-Br	*S*-ql-Br
CCDC number	2225250	2225243
Empirical formula	$C_{77}H_{73}AuBr_4Cl_2Cu_4F_6N_8O_{1.5}P$	$C_{77}H_{73}AuBr_4Cl_2Cu_4F_6N_8O_{1.5}P$
Formula weight	2121.07	2121.07
Temperature/K	200	200
Crystal system	orthorhombic	orthorhombic
Space group	$P2_12_12_1$	$P2_12_12_1$
a/Å	18.0354(11)	18.1049(10)
b/Å	19.6631(10)	19.6370(10)
c/Å	25.8766(15)	25.8205(14)
α/°	90	90
β/°	90	90
γ/°	90	90
Volume/Å^3	9176.7(9)	9179.9(9)
Z	4	4
ρ_{calc} g/cm^3	1.535	1.535
μ/mm^{-1}	4.378	4.376
F(000)	4164	4164
Crystal size/mm^3	0.12 × 0.04 × 0.04	0.12 × 0.04 × 0.03
Radiation	MoKα (λ = 0.71073)	MoKα (λ = 0.71073)
2θ range for data collection/°	3.768 to 55.124	3.776 to 55.004
Index ranges	$-23 \le h \le 23$, $-25 \le k \le 25$, $-33 \le l \le 33$	$-23 \le h \le 23$, $-25 \le k \le 25$, $-33 \le l \le 33$
Reflections collected	606321	642823
Independent reflections	21127 [R_{int} = 0.1304, R_{sigma} = 0.0361]	21052 [R_{int} = 0.0999, R_{sigma} = 0.0300]
Data/restraints/parameters	21127/116/1030	21052/61/962
Goodness-of-fit on F^2	1.107	1.099
Final R indexes [I>=2σ (I)]	R_1 = 0.0480, wR_2 = 0.1305	R_1 = 0.0433, wR_2 = 0.1196
Final R indexes [all data]	R_1 = 0.0587, wR_2 = 0.1381	R_1 = 0.0520, wR_2 = 0.1266
Largest diff. peak/hole / e Å^{-3}	1.43/-1.37	1.74/-1.11
Flack parameters	0.009(9)	0.004(2)

$R_1=\Sigma||F_o| - |F_c||/\Sigma|F_o|$, $wR_2=[\Sigma[w(F_o^2 - F_c^2)^2]/\Sigma w(F_o^2)^2]^{1/2}$.

Supplementary Table 6. Bond lengths in *R*-py-Br

bond lengths (Å)			
Au1-Cu1	2.8611(10)	Cu3-Br3	2.4428(14)
Au1-Cu2	2.8499(11)	Cu3-Br4	2.3875(14)
Au1-Cu3	2.9194(10)	Cu4-Br3	2.4130(13)
Au1-Cu4	2.8304(10)	Cu4-Br4	2.5279(15)
Au1-C5	2.028(7)	Cu1-Cu2	2.5069(14)
Au1-C20	2.033(7)	Cu3-Cu4	2.5621(14)
Cu1-Br1	2.4655(14)	Cu1-N1	1.958(7)
Cu2-Br1	2.4233(15)	Cu2-N2	1.961(7)
Cu1-Br2	2.4013(14)	Cu3-N3	1.982(6)
Cu2-Br2	2.4153(15)	Cu4-N4	1.983(7)

Supplementary Table 7. Bond lengths in *R*-py-I

bond lengths (Å)			
Au1-Cu1^{1}	2.8990(11)	Cu2-Cu2^{1}	2.4600(17)
Au1-Cu2	2.8985(11)	Cu1-Cu1^{1}	2.4617(17)
Au1-C4^{1}	2.052(5)	Cu1-N1	1.971(6)
Cu1^{1}-I1	2.5714(11)	Cu2-N4	1.970(6)
Cu1-I1	2.6042(10)	Cu2^{1}-I2	2.6036(10)
Cu2-I2	2.5719(11)		

Symmetry codes: ^{1}1 - X, 1 - Y, + Z.

Supplementary Table 8. Bond lengths in *R*-ql-Cl

bond lengths (Å)			
Au1-Cu1^{1}	2.8714(4)	Cu2-Cu2^{1}	2.5210(11)
Au1-Cu2^{1}	2.8561(5)	Cu1-N1	1.952(3)
Au1-C2^{1}	2.047(4)	Cu2-Cl2^{1}	2.3358(16)
Cu1-Cu1^{1}	2.4574(9)	Cu2-Cl2	2.3508(17)
Cu1-Cl1	2.2641(17)	Cu2-N4	1.951(4)
Cu1-Cl1^{1}	2.4034(17)		

Symmetry codes: ^{1}1 - X, + Y, 1/2 - Z.

433 **Supplementary Table 9.** Bond lengths in *R*-ql-Br

bond lengths (Å)			
Au1-Cu1	2.9084(16)	Cu3-Br3	2.4524(19)
Au1-Cu2	2.8647(14)	Cu3-Br4	2.4561(19)
Au1-Cu3	2.8610(13)	Cu4-Br3	2.4488(18)
Au1-Cu4	2.9019(14)	Cu4-Br4	2.4640(19)
Au1-C9	2.008(9)	Cu1-Cu2	2.5823(19)
Au1-C21	2.029(10)	Cu3-Cu4	2.6162(17)
Cu1-Br1	2.429(3)	Cu1-N1	2.000(10)
Cu2-Br1	2.5031(19)	Cu2-N2	1.984(8)
Cu1-Br2	2.449(2)	Cu3-N3	1.993(8)
Cu2-Br2	2.404(2)	Cu4-N4	1.979(8)

434

435 **Supplementary Table 10.** Bond lengths in *R*-ql-I

bond lengths (Å)			
Au1-Cu1	2.9816(8)	Cu3-I3	2.5746(10)
Au1-Cu2	3.0037(7)	Cu3-I4	2.6322(10)
Au1-Cu3	2.9194(7)	Cu4-I3	2.6420(10)
Au1-Cu4	3.0191(7)	Cu4-I4	2.6036(10)
Au1-C7	2.043(5)	Cu1-Cu2	2.4305(9)
Au1-C8	2.046(5)	Cu3-Cu4	2.4289(9)
Cu1-I1	2.5673(10)	Cu1-N1	1.980(4)
Cu2-I1	2.6048(11)	Cu2-N2	1.984(4)
Cu1-I2	2.6214(10)	Cu3-N3	1.986(5)
Cu2-I2	2.5816(10)	Cu4-N4	1.978(5)

436

Supplementary Table 11. Crystal data and structure refinement for *R*-ql-I at different temperatures

	100 K	150 K
CCDC number	2225244	2225246
Empirical formula	$C_{74}H_{66}AuCu_4F_6I_4N_8OP$	$C_{74}H_{66}AuCu_4F_6I_4N_8OP$
Formula weight	2187.04	2187.04
Temperature/K	100	150
Crystal system	orthorhombic	orthorhombic
Space group	$P2_12_12$	$P2_12_12$
a/Å	19.4472(9)	26.6715(11)
b/Å	26.6351(14)	19.4832(8)
c/Å	14.7879(8)	14.8453(7)
α/°	90	90
β/°	90	90
γ/°	90	90
Volume/Å^3	7659.8(7)	7714.3(6)
Z	4	4
ρ_{calc} g/cm^3	1.896	1.883
μ/mm^{-1}	4.697	4.664
F(000)	4200	4200
Crystal size/mm^3	0.12 × 0.04 × 0.04	0.12 × 0.04 × 0.04
Radiation	MoKα (λ = 0.71073)	MoKα (λ = 0.71073)
2θ range for data collection/°	3.782 to 55.024	3.772 to 54.966
Index ranges	$-25 \leq h \leq 25$, $-34 \leq k \leq 34$, $-19 \leq l \leq 19$	$-34 \leq h \leq 34$, $-25 \leq k \leq 25$, $-19 \leq l \leq 19$
Reflections collected	356146	405554
Independent reflections	17579 [R_{int} = 0.0938, R_{sigma} = 0.0306]	17700 [R_{int} = 0.0901, R_{sigma} = 0.0270]
Data/restraints/parameters	17579/13/889	17700/9/888
Goodness-of-fit on F^2	1.149	1.080
Final R indexes [I>=2σ (I)]	R_1 = 0.0229, wR_2 = 0.0558	R_1= 0.0240, wR_2 = 0.0616
Final R indexes [all data]	R_1 = 0.0266, wR_2 = 0.0628	R_1= 0.0285, wR_2 = 0.0636
Largest diff. peak/hole / e Å^{-3}	1.80/-1.74	1.46/-1.62
Flack parameters	0.010(4)	0.0013(17)

Supplementary Table 11 (continued)

	180 K	250 K
CCDC number	2225245	2225248
Empirical formula	$C_{74}H_{66}AuCu_4F_6I_4N_8OP$	$C_{74}H_{66}AuCu_4F_6I_4N_8OP$
Formula weight	2187.04	2187.04
Temperature/K	180	250.0
Crystal system	orthorhombic	orthorhombic
Space group	$P2_12_12$	$P2_12_12$
a/Å	26.6989(12)	26.7394(12)
b/Å	19.5129(8)	19.5607(8)
c/Å	14.8876(7)	14.9925(7)
α/°	90	90
β/°	90	90
γ/°	90	90
Volume/Å^3	7756.0(6)	7841.7(6)
Z	4	4
ρ_{calc} g/cm^3	1.873	1.852
μ/mm^{-1}	4.638	4.588
F(000)	4200	4200
Crystal size/mm^3	0.12 × 0.04 × 0.04	0.12 × 0.04 × 0.04
Radiation	MoKα (λ = 0.71073)	MoKα (λ = 0.71073)
2θ range for data collection/°	3.764 to 55.208	3.746 to 55.014
Index ranges	$-34 \leq h \leq 34$, $-25 \leq k \leq 25$, $-19 \leq l \leq 19$	$-34 \leq h \leq 34$, $-25 \leq k \leq 25$, $-19 \leq l \leq 19$
Reflections collected	580981	417573
Independent reflections	17885 [R_{int} = 0.0900, R_{sigma} = 0.0238]	18007 [R_{int} = 0.0970, R_{sigma} = 0.0299]
Data/restraints/parameters	17885/19/890	18007/32/887
Goodness-of-fit on F^2	1.104	1.095
Final R indexes [I>=2σ (I)]	R_1 = 0.0231, wR_2 = 0.0592	R_1 = 0.0287, wR_2 = 0.0630
Final R indexes [all data]	R_1 = 0.0298, wR_2 = 0.0643	R_1 = 0.0418, wR_2 = 0.0728
Largest diff. peak/hole / e Å^{-3}	1.21/-1.59	1.00/-1.64
Flack parameters	0.0031(15)	-0.002(2)

Supplementary Table 11 (continued)

	300 K
CCDC number	2225249
Empirical formula	$C_{70}H_{56}AuI_4Cu_4F_6N_8P$
Formula weight	2112.92
Temperature/K	300.0
Crystal system	orthorhombic
Space group	$P2_12_12$
a/Å	19.5906(6)
b/Å	26.7630(8)
c/Å	15.0553(5)
α/°	90
β/°	90
γ/°	90
Volume/Å^3	7893.5(4)
Z	4
ρ_{calc} g/cm^3	1.778
μ/mm^{-1}	4.553
F(000)	4032
Crystal size/mm^3	0.12 × 0.04 × 0.04
Radiation	MoKα (λ = 0.71073)
2θ range for data collection/°	3.736 to 55.042
Index ranges	$-25 \le h \le 25$, $-34 \le k \le 34$, $-19 \le l \le 19$
Reflections collected	466984
Independent reflections	18167 [R_{int} = 0.1224, R_{sigma} = 0.0337]
Data/restraints/parameters	18167/71/889
Goodness-of-fit on F^2	1.071
Final R indexes [I>=2σ (I)]	R_1 = 0.0375, wR_2 = 0.0906
Final R indexes [all data]	R_1 = 0.0606, w R_2 = 0.1042
Largest diff. peak/hole / e Å^{-3}	1.13/-1.78
Flack parameters	0.007(6)

$R_1=\Sigma||F_o| - |F_c||/\Sigma|F_o|$, $wR_2=[\Sigma[w(F_o^2 - F_c^2)^2]/\Sigma w(F_o^2)^2]^{1/2}$.

Supplementary Table 12. Cu-Cu bond lengths for *R*-ql-I at different temperatures.

	100 K	150 K	180 K	200 K	250 K	300 K
Cu1-Cu2	2.4236(9)	2.4264(9)	2.4265(9)	2.4305(9)	2.4327(12)	2.4356(14)
Cu3-Cu4	2.4152(9)	2.4214(9)	2.4281(9)	2.4289(10)	2.4325(11)	2.4363(16)

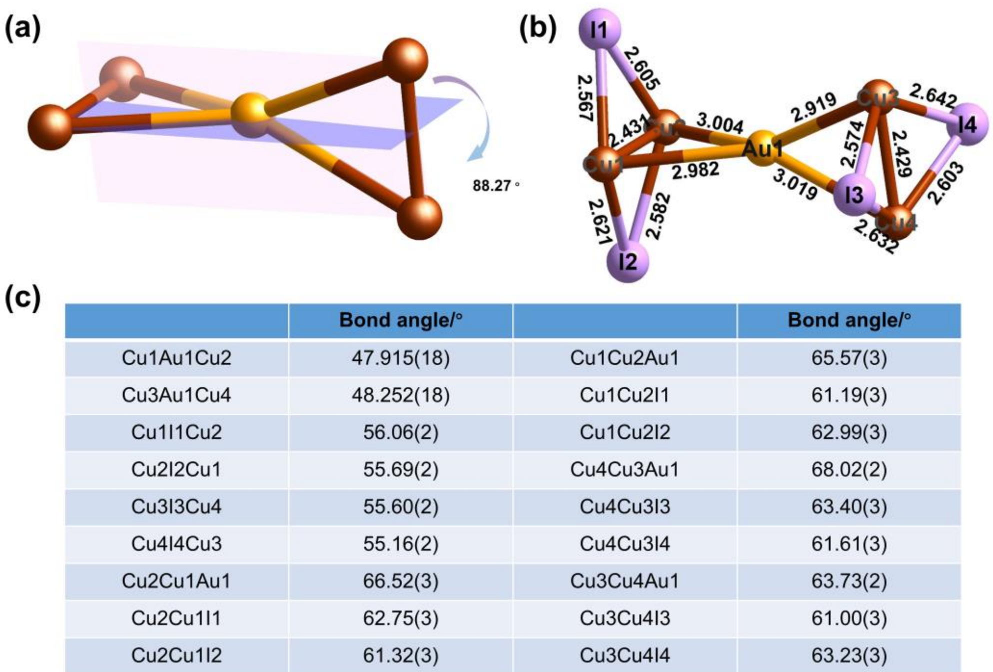

(c)

	Bond angle/°		Bond angle/°
Cu1Au1Cu2	47.915(18)	Cu1Cu2Au1	65.57(3)
Cu3Au1Cu4	48.252(18)	Cu1Cu2I1	61.19(3)
Cu1I1Cu2	56.06(2)	Cu1Cu2I2	62.99(3)
Cu2I2Cu1	55.69(2)	Cu4Cu3Au1	68.02(2)
Cu3I3Cu4	55.60(2)	Cu4Cu3I3	63.40(3)
Cu4I4Cu3	55.16(2)	Cu4Cu3I4	61.61(3)
Cu2Cu1Au1	66.52(3)	Cu3Cu4Au1	63.73(2)
Cu2Cu1I1	62.75(3)	Cu3Cu4I3	61.00(3)
Cu2Cu1I2	61.32(3)	Cu3Cu4I4	63.23(3)

Supplementary Figure 5. (a) Motif of $AuCu_4$ in *R*-ql-I. (b) Distorted structure of the $AuCu_4I_4$ skeleton in *R*-ql-I, including bond length. (c) The bond angle of the $AuCu_4I_4$ skeleton in *R*-ql-I. C olor codes: Au, yellow; I, purple; Cu, brown.

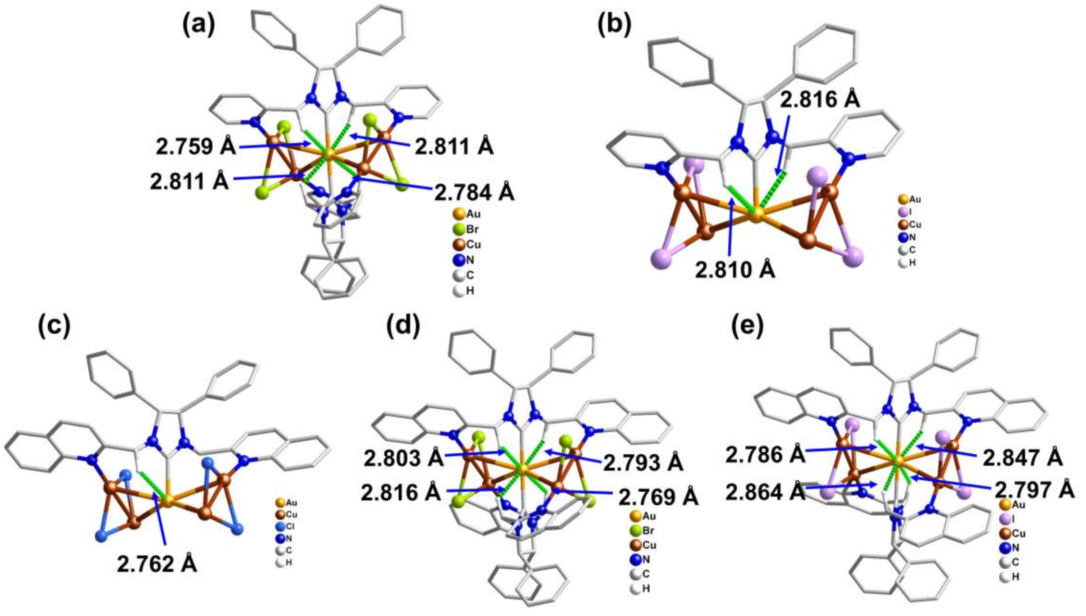

Supplementary Figure 6. Structure motifs and the corresponding schemes showing intramolecular C–H ···Au interactions in (a) *R*-py-Br, (b) *R*-py-I, (c) *R*-ql-Cl, (d) *R*-ql-Br, and (e) *R*-ql-I.

10.4. Characterization of clusters

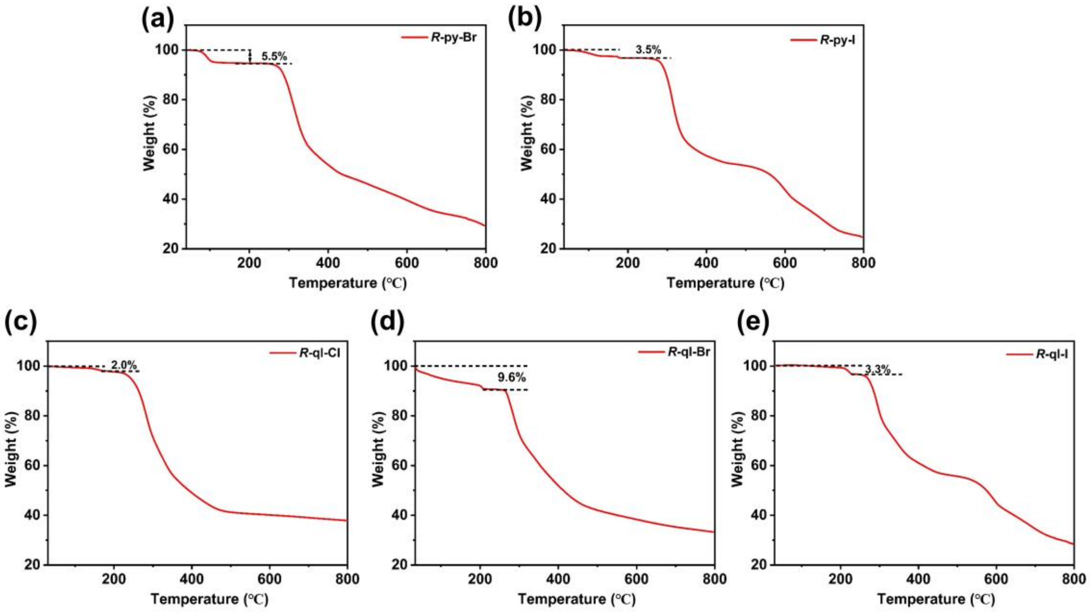

Supplementary Figure 7. TG curves of (a) *R*-py-Br, (b) *R*-py-I, (c) *R*-ql-Cl, (d) *R/S*-ql-Br, and (e) *R*-ql-I under nitrogen atmosphere. For *R*-py-Br, the weight loss of solvent molecules ($(C_2H_5)_2O$ and 0.5 CH_2Cl_2) reaches approximately 5.5%, which coincides with the calculated result (5.7%). For *R*-py-I, the weight loss of solvent molecules ($(C_2H_5)_2O$) is about 3.5%, which is consistent with the calculated result (3.7%). For *R*-ql-Cl, the weight loss of solvent molecules (0.5 $(C_2H_5)_2O$) reaches approximately 2.0%, which is consistent with the calculated result (2.0%). For *R*-ql-Br, the weight loss of solvent molecules (1.5 $(C_2H_5)_2O$ and CH_2Cl_2) is about 9.6%, which coincides with the calculated result (9.7%). For *R*-ql-I, the weight loss of solvent molecules ($(C_2H_5)_2O$) reaches approximately 3.3%, which coincides with the calculated result (3.4%).

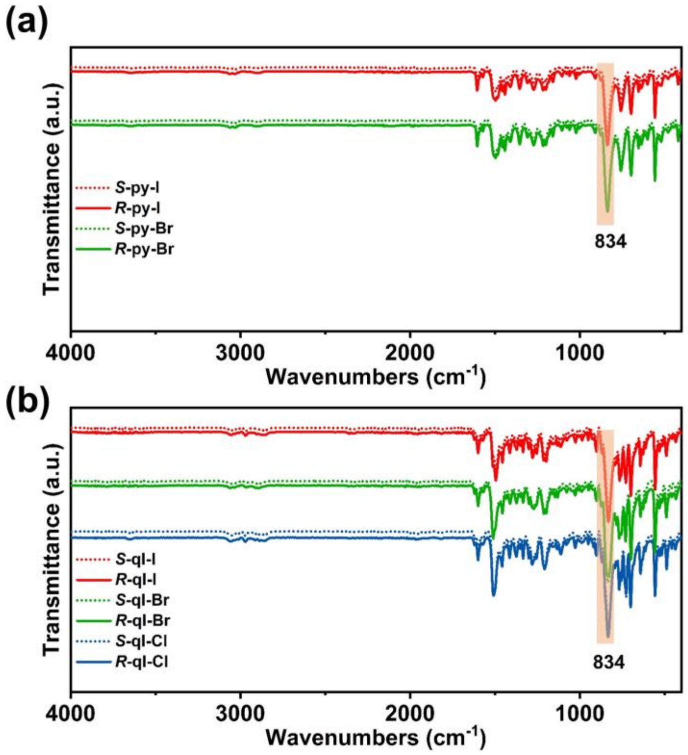

Supplementary Figure 8. FT-IR spectra of (a) *R/S*-py-Br (green line), *R/S*-py-I (red line), (b) *R/S*-ql-Cl (blue line), *R/S*-ql-Br (green line), *R/S*-ql-I (red line). The bands at 834 cm^{-1} are attributable to the PF_6^- anion.

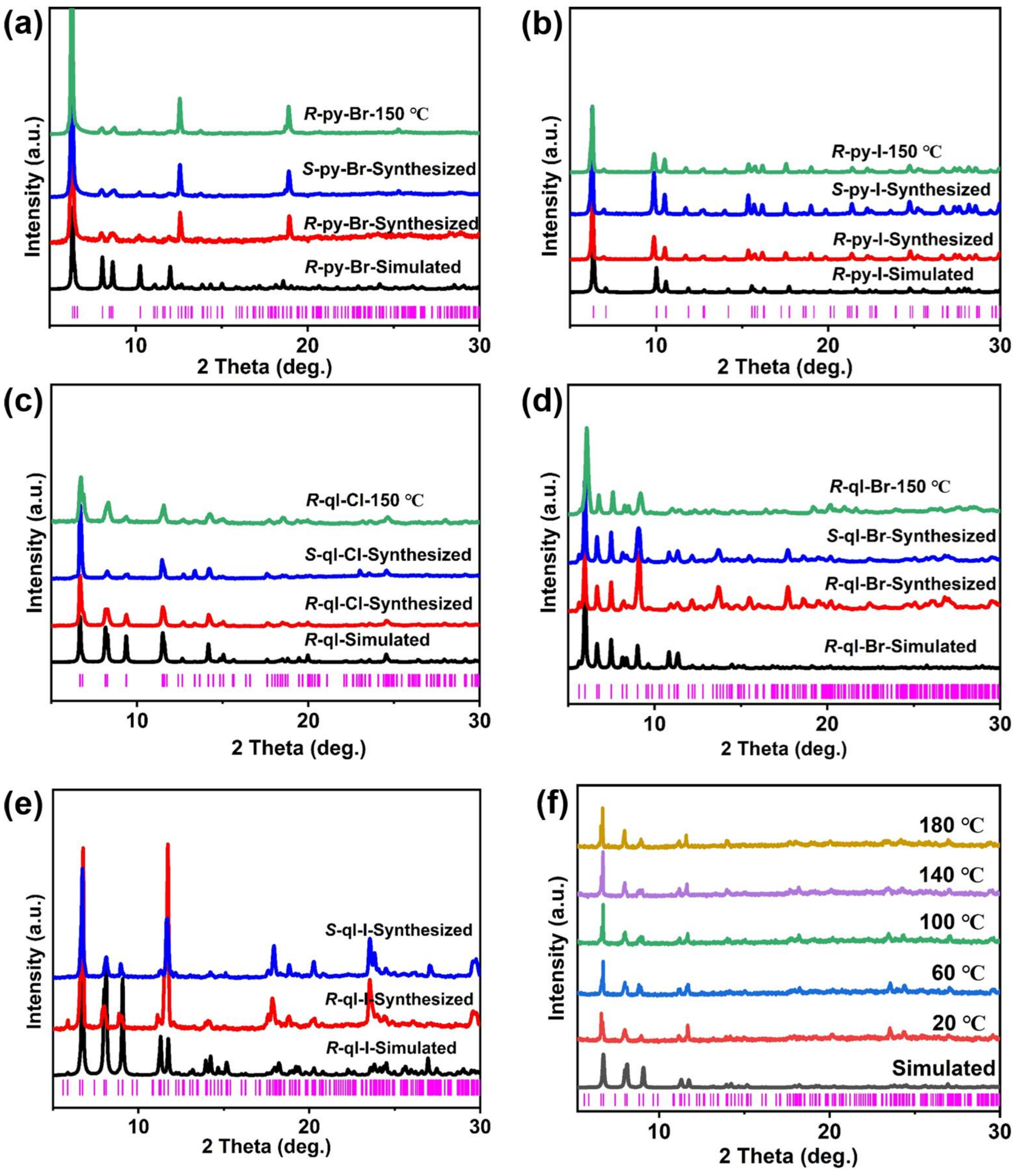

Supplementary Figure 9. PXRD patterns of (a) *R/S*-py-Br, (b) *R/S*-py-I, (c) *R/S*-ql-Cl, (d) *R/S*-ql-Br, (e,f) *R/S*-ql-I.

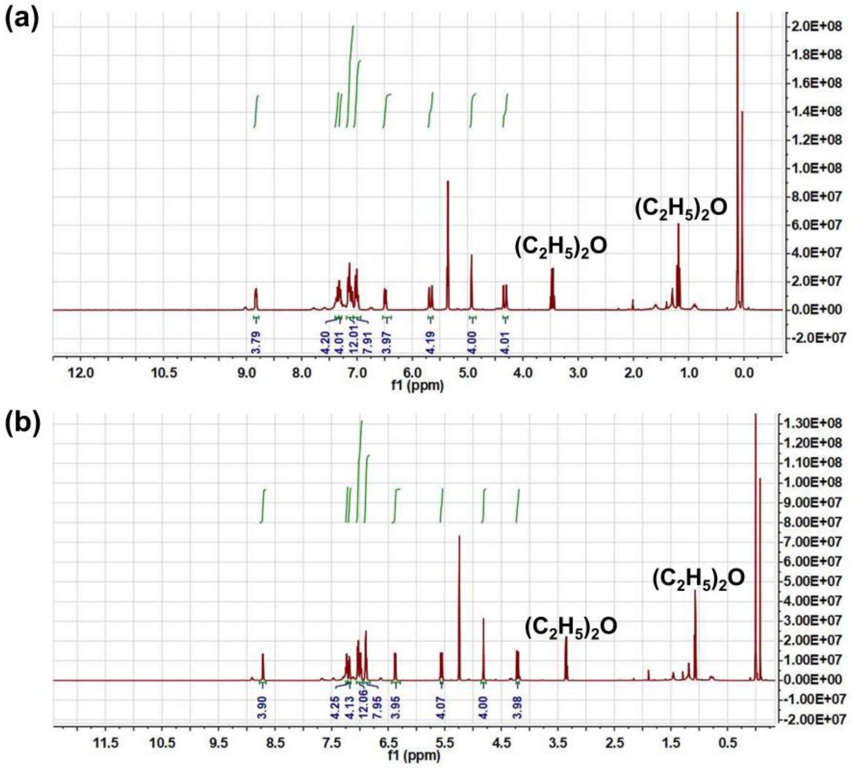

Supplementary Figure 10. ^{1}H NMR spectra of (a) *R*-py-Br and (b) *S*-py-Br (600 MHz, CD_2Cl_2).

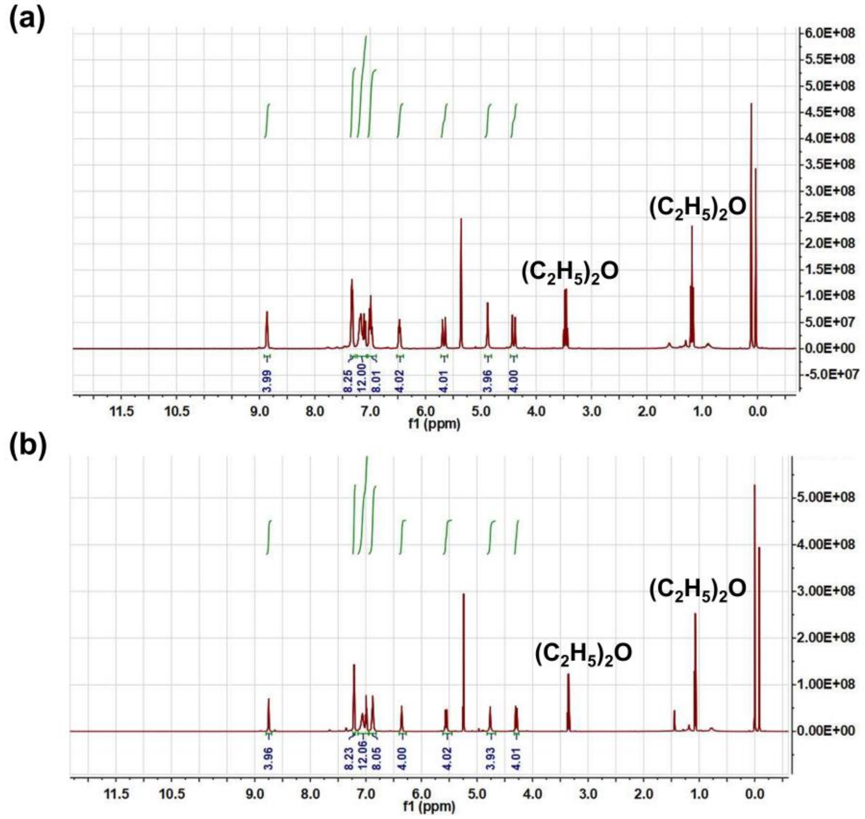

Supplementary Figure 11. ^{1}H NMR spectra of (a) *R*-py-I and (b) *S*-py-I (600 MHz, CD_2Cl_2).

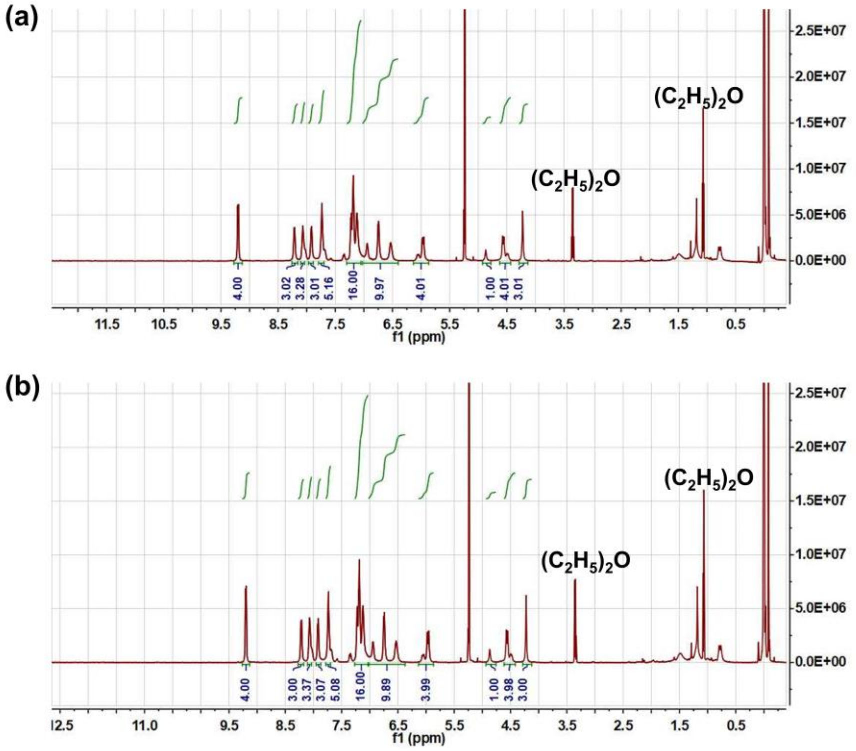

Supplementary Figure 12. ^{1}H NMR spectra of (a) *R*-ql-Cl and (b) *S*-ql-Cl (600 MHz, CD_2Cl_2).

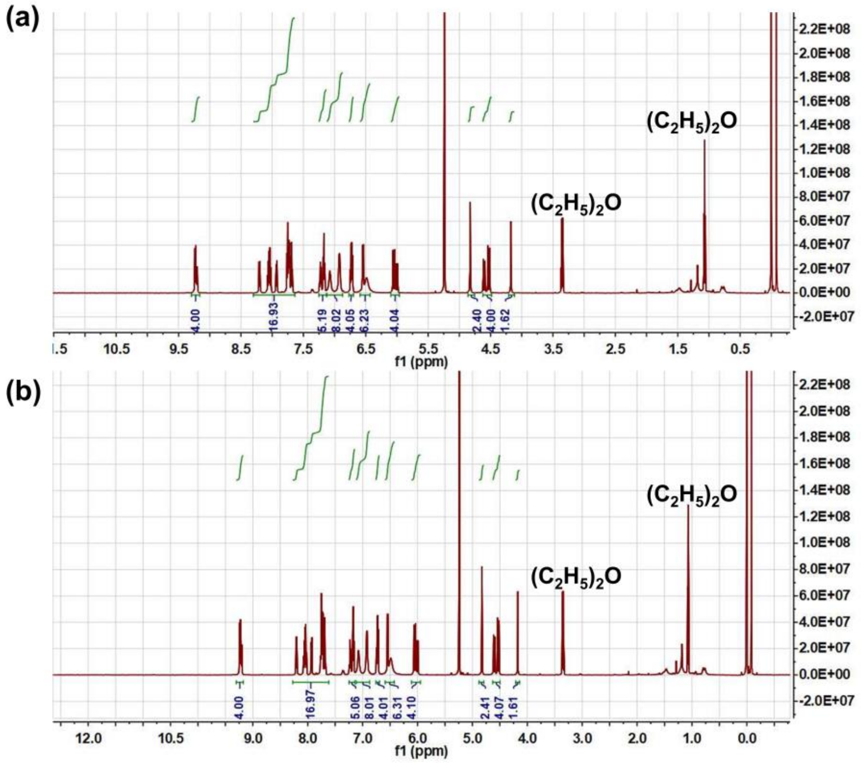

Supplementary Figure 13. ^{1}H NMR spectra of (a) *R*-ql-Br and (b) *S*-ql-Br (600 MHz, CD_2Cl_2).

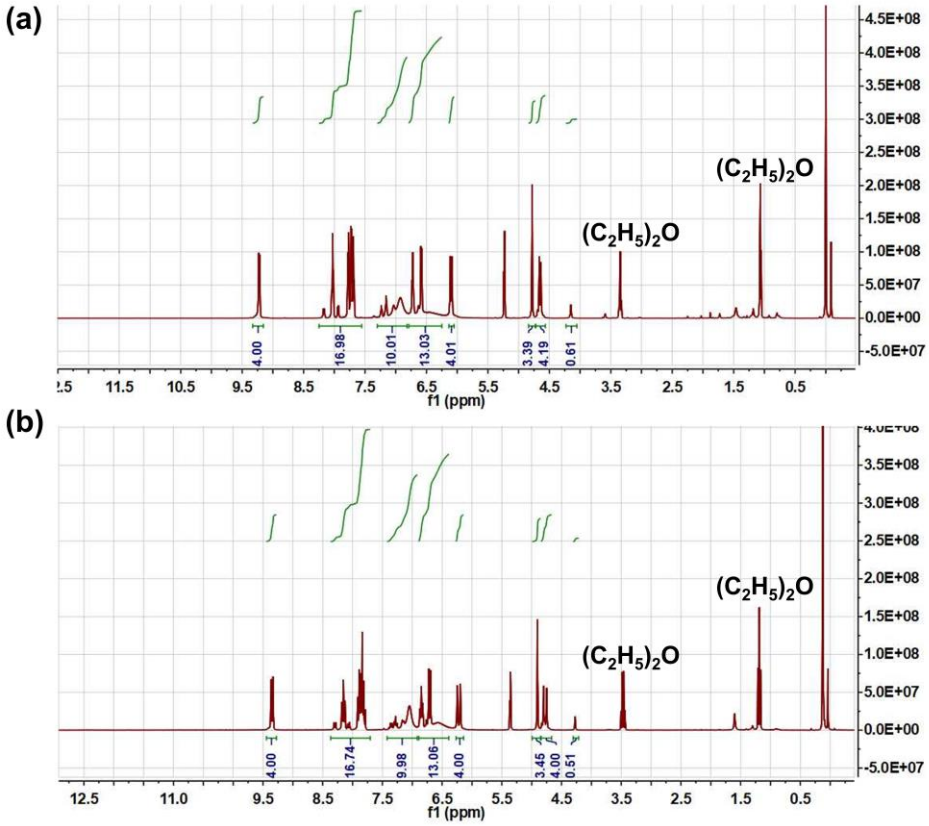

Supplementary Figure 14. ^{1}H NMR spectra of (a) *R*-ql-I and (b) *S*-ql-I (600 MHz, CD_2Cl_2).

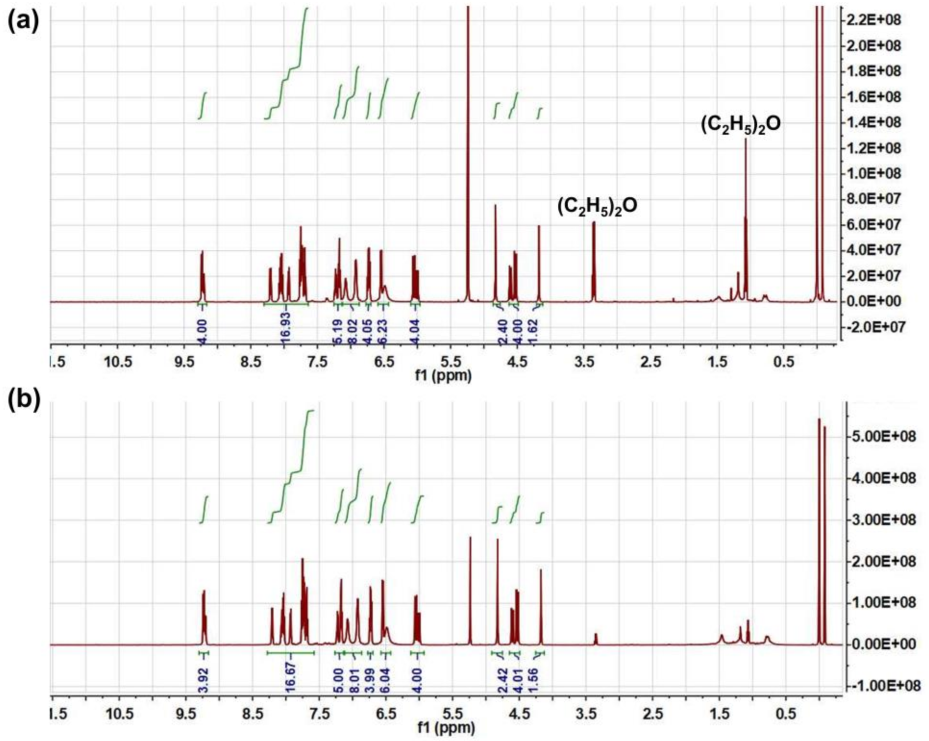

Supplementary Figure 15. (a) ^{1}H-NMR spectrum of the *R*-ql-Br cluster. (b) ^{1}H-NMR spectrum of the *R*-ql-Br cluster after heat treatment at 150 °C (600 MHz, CD_2Cl_2).

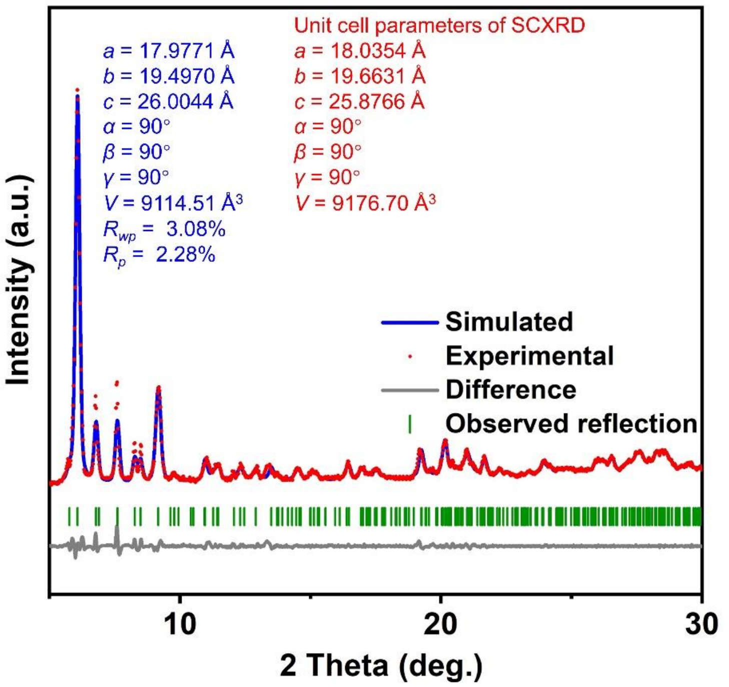

Supplementary Figure 16. Pawley refinement of the PXRD pattern for *R*-ql-Br cluster after heat treatment at 150 ℃.

10.5. Luminescence spectra of clusters

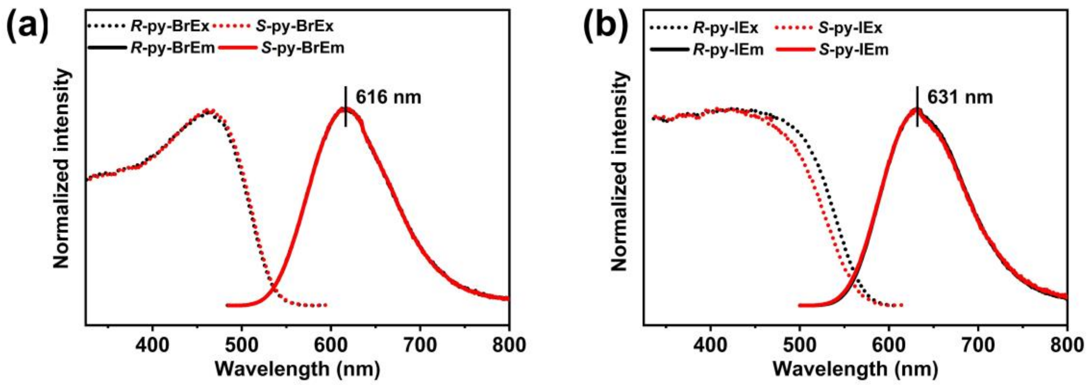

Supplementary Figure 17. Excitation-emission spectra of (a) *R/S*-py-Br, (b) *R/S*-py-I in the solid state.

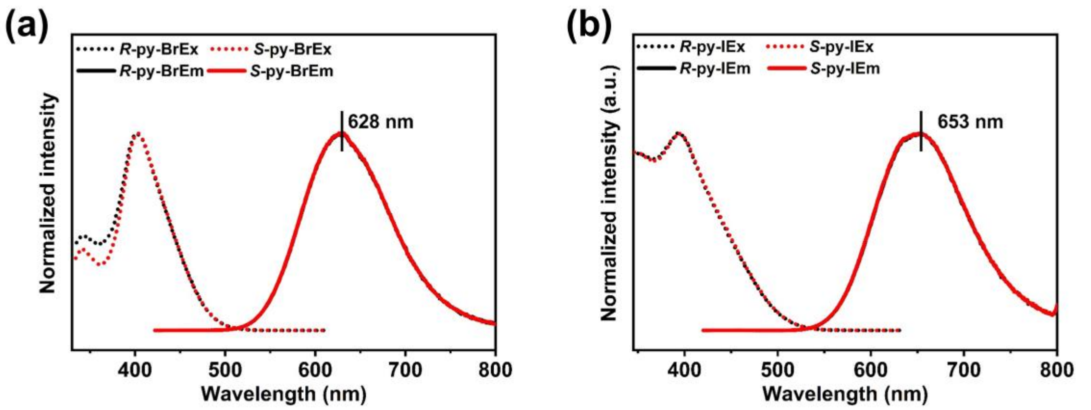

Supplementary Figure 18. Excitation-emission spectra of (a) *R/S*-py-Br, (b) *R/S*-py-I in CH_2Cl_2 (1×10^{-5} mol/L) under ambient conditions.

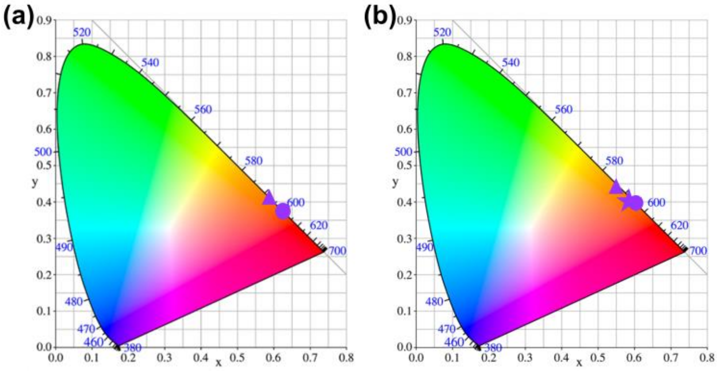

(c)

	CIE
▲ *R*-py-Br	(0.59,0.41)
● *R*-py-I	(0.63,0.37)

(d)

	CIE
★ *R*-ql-Cl	(0.59,0.40)
▲ *R*-ql-Br	(0.56,0.44)
● *R*-ql-I	(0.60,0.40)

Supplementary Figure 19. (a,c) The CIE coordinates of *R*-py-Br and *R*-py-I. (b,d) The CIE coordinates of *R*-ql-Cl, *R*-ql-Br and *R*-ql-I.

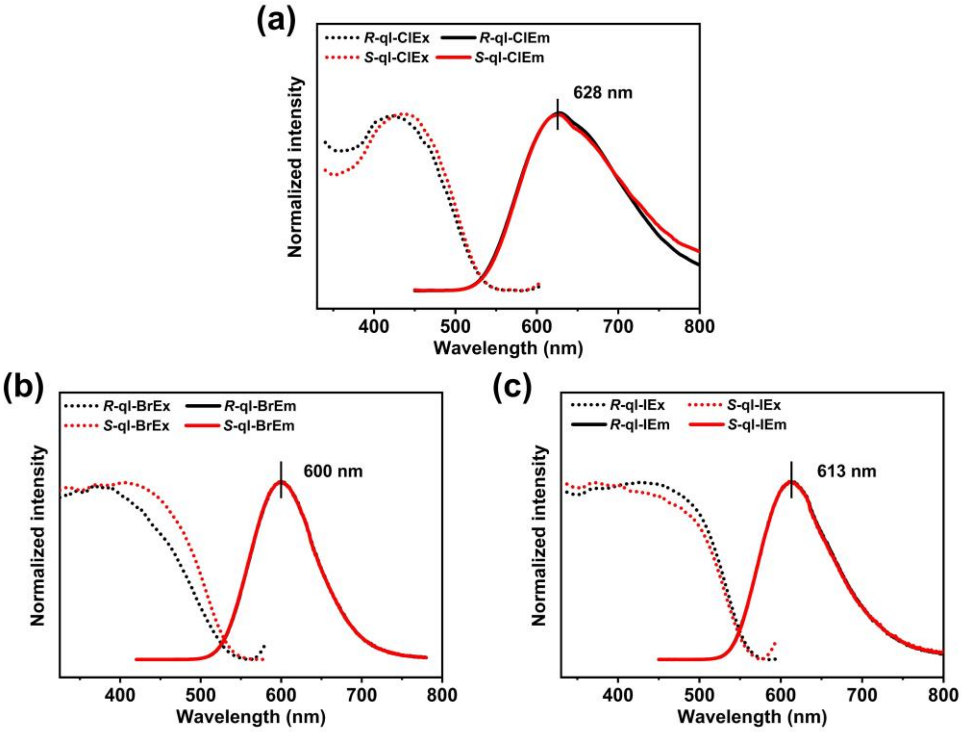

Supplementary Figure 20. Excitation-emission spectra of (a) *R/S*-ql-Cl, (b) *R/S*-ql-Br (c) *R/S*-ql-I in the solid state.

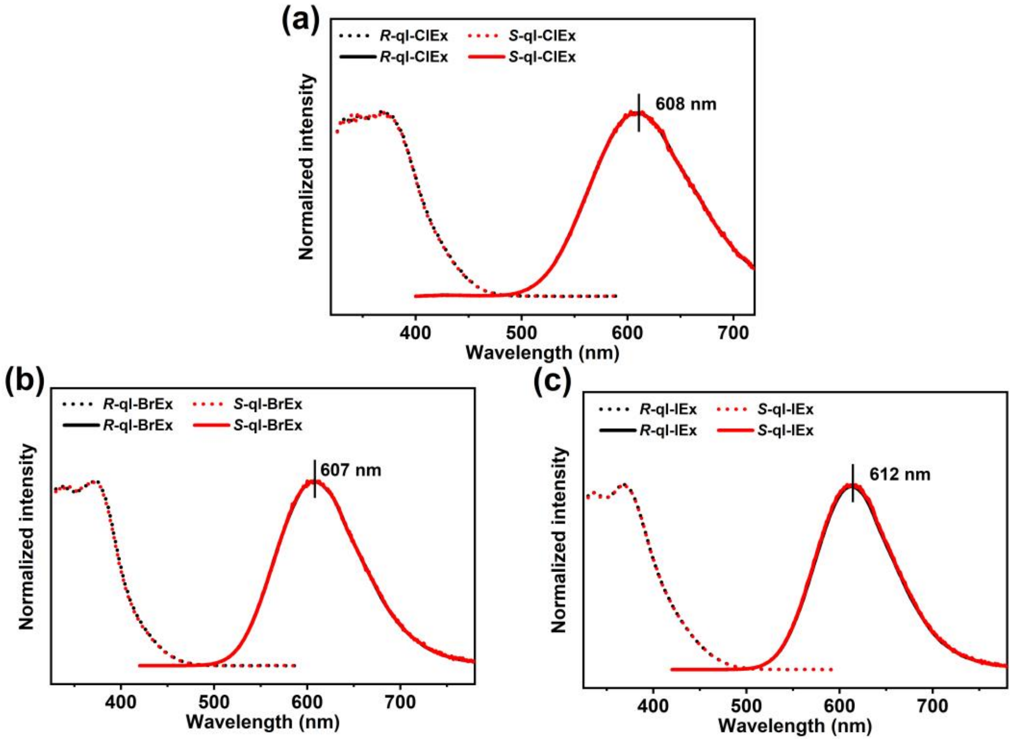

Supplementary Figure 21. Excitation-emission spectra of (a) *R/S*-NHC^{ql}-$AuCu_4$-Cl, (b) *R/S*-NHC^{ql}-$AuCu_4$-Br (c) *R/S*-NHC^{ql}-$AuCu_4$-I in CH_2Cl_2 (1×10^{-5} mol/L) under ambient conditions.

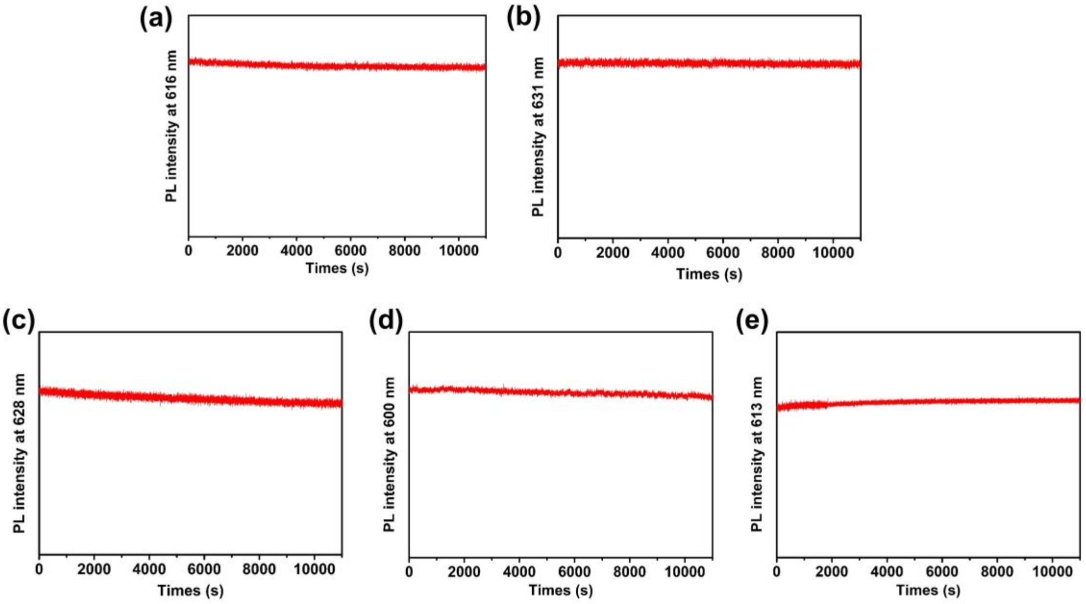

Supplementary Figure 22. Time-dependent fluorescence spectra of (a) *R*-py-Br, (b) *R*-py-I, (c) *R*-ql-Cl, (d) *R*-ql-Br, (e) *R*-ql-I exposed to 400 nm UV light for 3 h in the solid state.

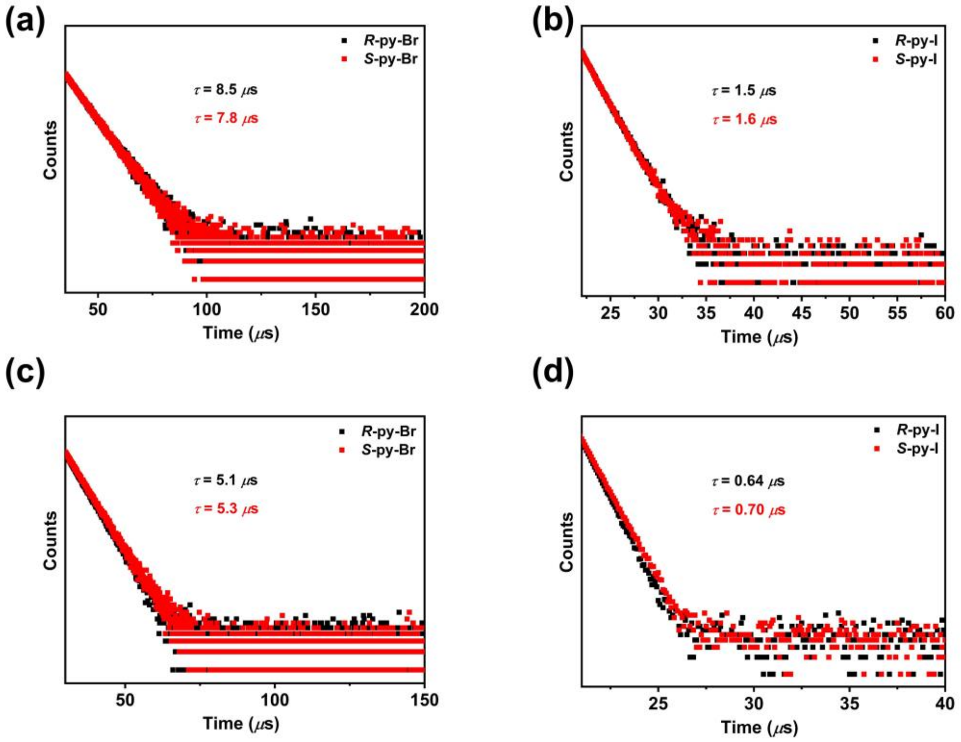

Supplementary Figure 23. Lifetimes of (a) *R/S*-py-Br, (b) *R/S*-py-I in the solid-state. Lifetimes of (c) *R/S*-py-Br, (d) *R/S*-py-I in CH_2Cl_2 (1×10^{-5} mol/L) under ambient conditions.

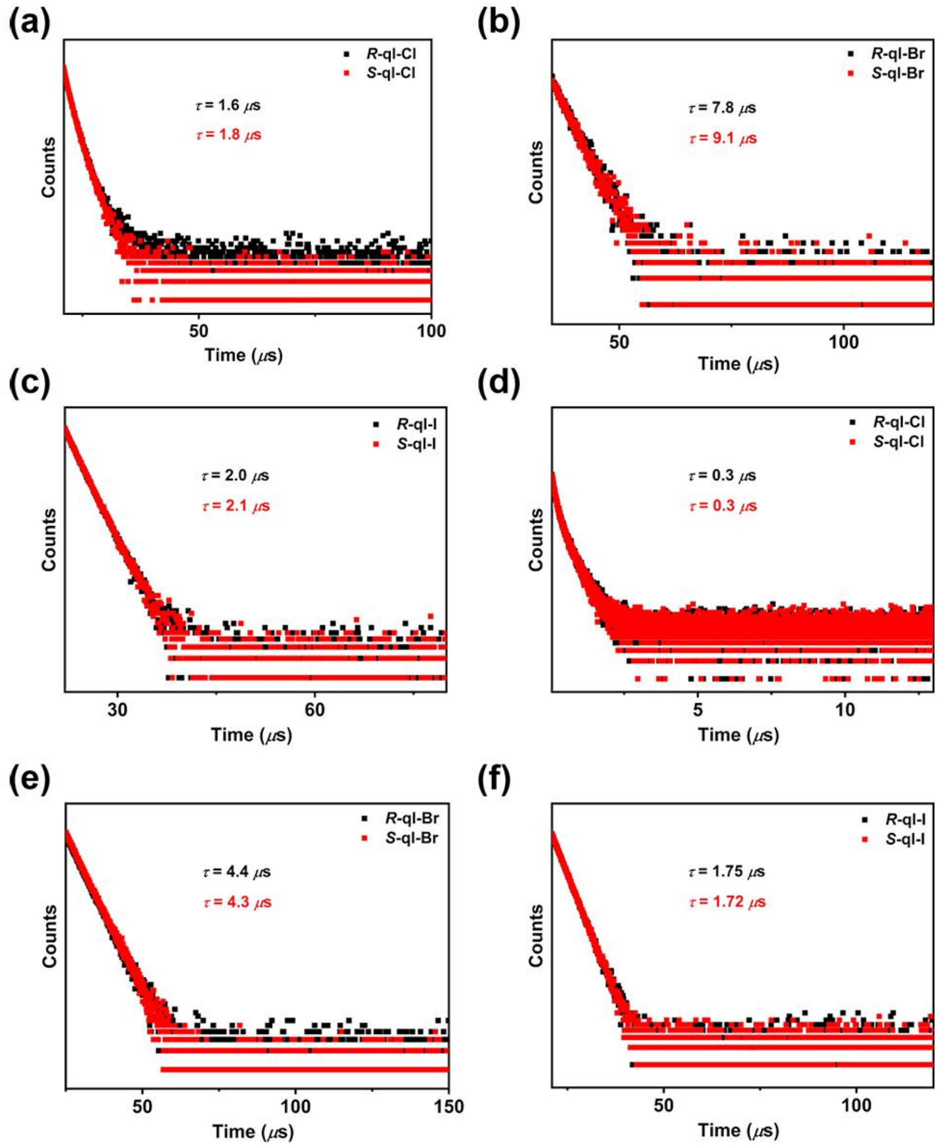

Supplementary Figure 24. Lifetimes of (a) *R/S*-ql-Cl, (b) *R/S*-ql-Br (c) *R/S*-ql-I in the solid-state. Lifetimes of (d) *R/S*-ql-Cl, (e) *R/S*-ql-Br (f) *R/S*-ql-I in CH_2Cl_2 (1×10^{-5} mol/L) under ambient conditions.

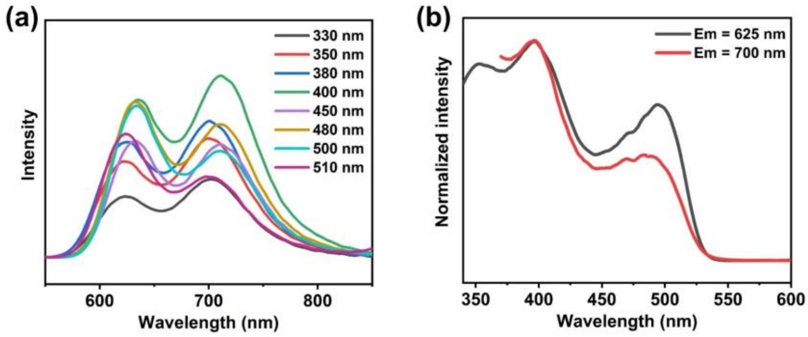

Supplementary Figure 25. (a) Solid-state emission spectra of *R*-ql-I at different excitation wavelengths at 93 K. (b) Solid-state excitation spectra of *R*-ql-I at different emission wavelengths at 93 K.

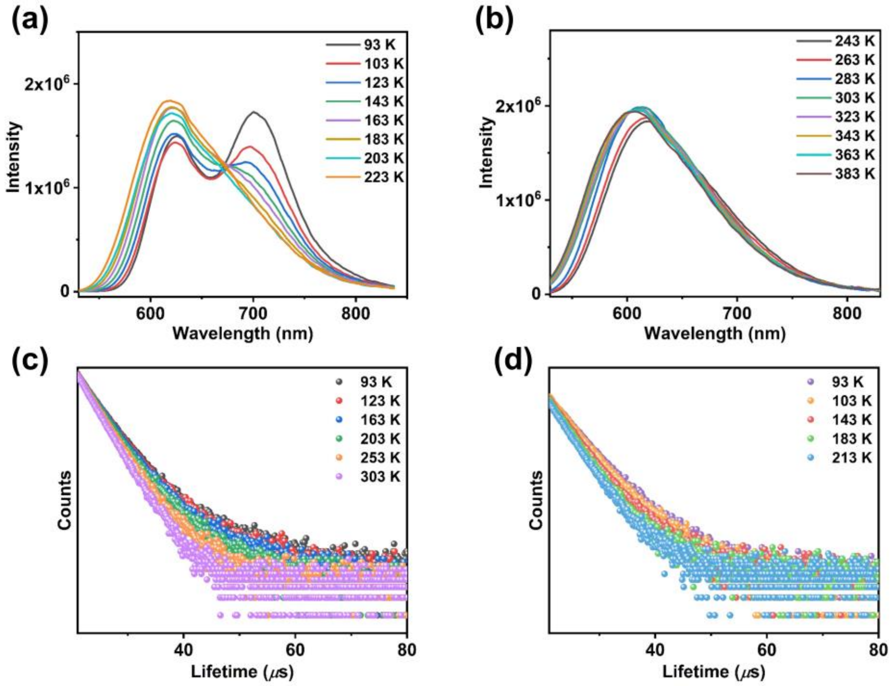

Supplementary Figure 26. Temperature-dependent solid-state emission spectra of *R*-ql-I under excitation at 400 nm. (a) 93-223 K. (b) 243-383 K. Temperature dependence of the excited-state lifetimes for an emission wavelength (c) of 625 nm in the range of 93 to 303 K and (d) of 700 nm in the range of 93 to 183 K.

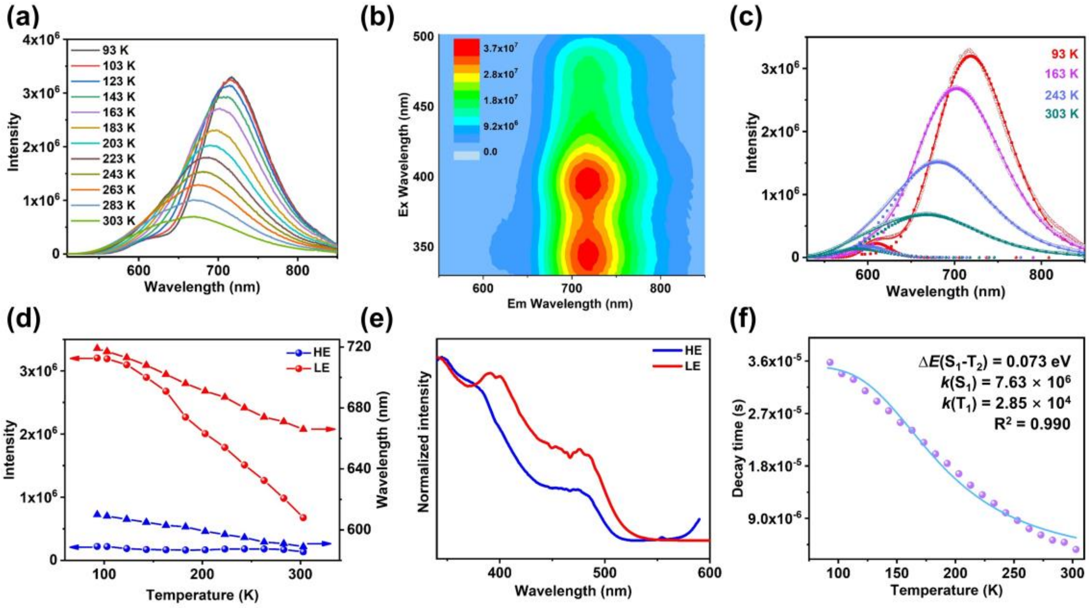

Supplementary Figure 27. (a) Temperature-dependent solid-state emission spectra of *R*-ql-Cl under excitation at 400 nm in the range of 93 to 303 K. (b) Three-dimensional excitation-emission matrix (3D-EX-EM) luminescence spectra of *R*-ql-Cl in the solid-state at 93 K. (c) Emission spectra of *R*-ql-Cl fitted by the combination of Gaussian shaped HE (square) and LE (solid sphere) emissions at different temperature; the hollow circles represent the original data, and the solid lines represent the fitted data. (d) The maximum emission wavelength and intensity of *R*-ql-Cl at LE and HE at different temperatures. (e) Solid-state excitation spectra of *R*-ql-Cl at different emission wavelengths at 93 K. (f) Plot of emission decay lifetime at LE against temperature (93 to 303 K); the blue line represents the fit according to the TADF equation.

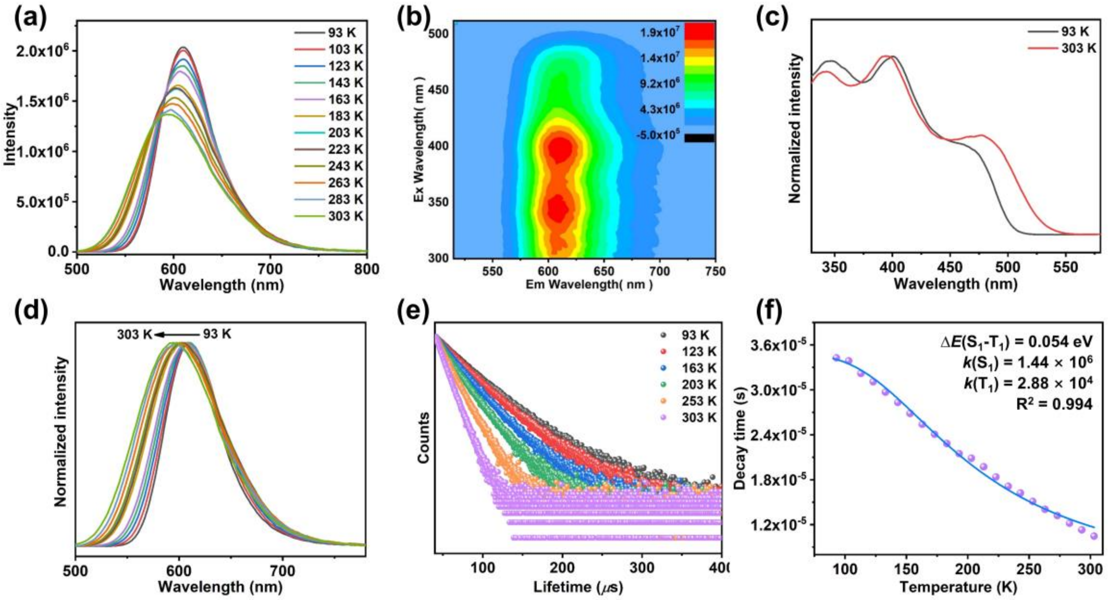

Supplementary Figure 28. Non-normalized (a) and normalized (d) temperature-dependent solid-state emission spectra of *R*-ql-Br under excitation at 400 nm in the range of 93 to 303 K. (b) 3D-EX-EM luminescence spectra of *R*-ql-Br in the solid-state at 93 K. (c) Solid-state excitation spectra of *R*-ql-Br at 93 and 303 K. (e) Plot of emission decay lifetime against temperature (93 to 303 K); the blue line represents the fit according to the TADF equation. (f) Temperature dependence of the excited-state lifetimes in the range of 93 to 303 K.

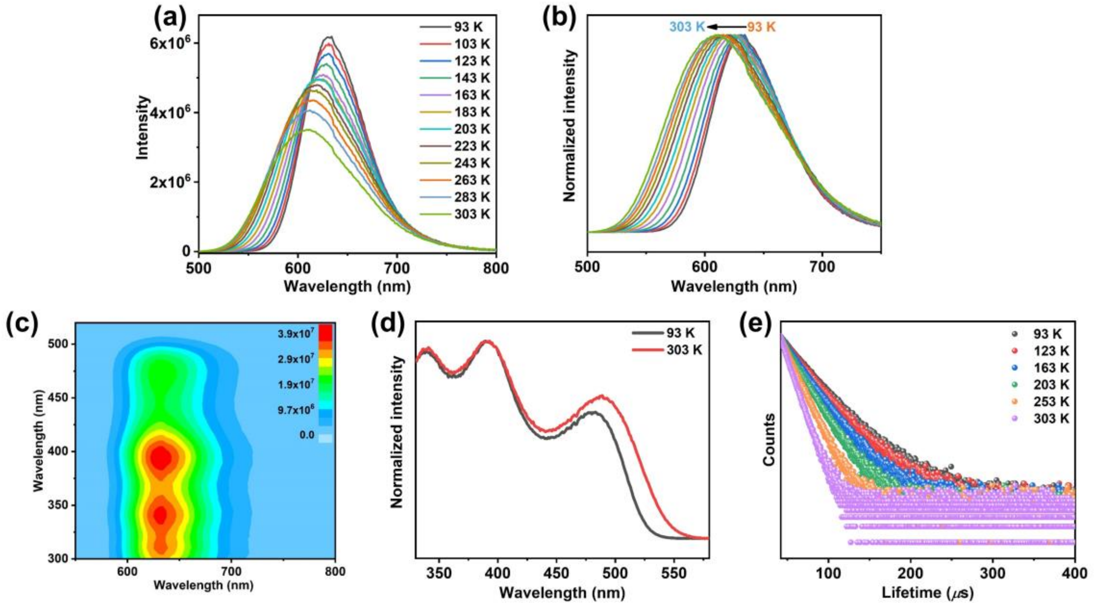

Supplementary Figure 29. Non-Normalized (a) and normalized (b) temperature-dependent solid-state emission spectra of *R*-py-Br under excitation at 400 nm in the range of 93 to 303 K. (c) 3D-EX-EM luminescence spectra of *R*-py-Br in the solid-state at 93 K. (d) Solid-state excitation spectra of *R*-py-Br at 93 and 303 K. (e) Temperature dependence of the excited-state lifetimes in the range of 93 to 303 K.

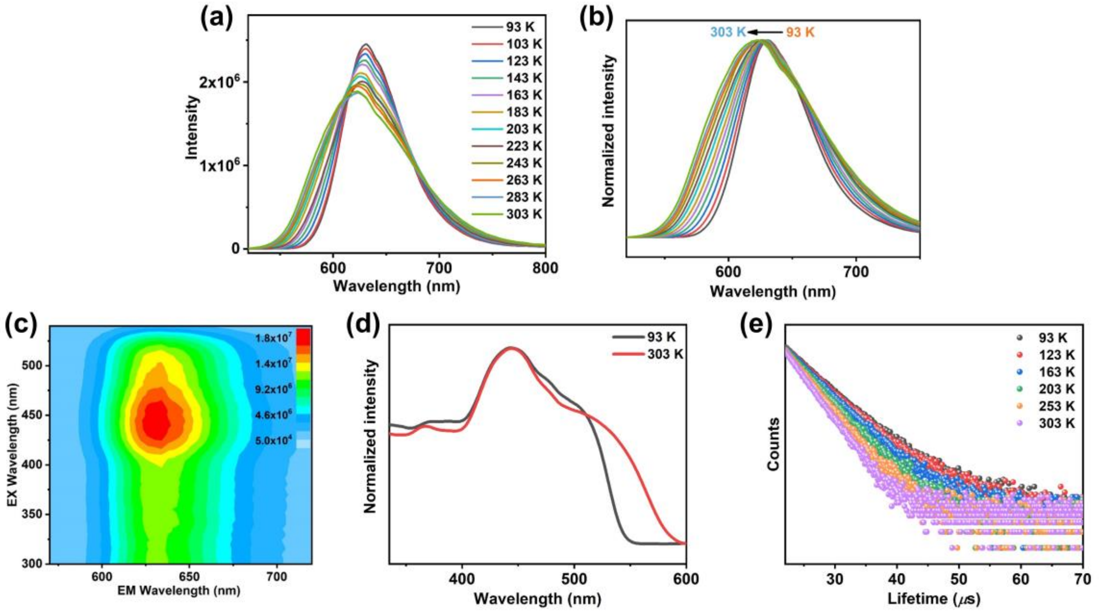

Supplementary Figure 30. Non-Normalized (a) and normalized (b) temperature-dependent solid-state emission spectra of *R*-py-I under excitation at 400 nm in the range of 93 to 303 K. (c) 3D-EX-EM luminescence spectra of *R*-py-I in the solid-state at 93 K. (d) Solid-state excitation spectra of *R*-py-I at 93 and 303 K. (e) Temperature dependence of the excited-state lifetimes in the range of 93 to 303 K.

10.6. Theoretical calculations

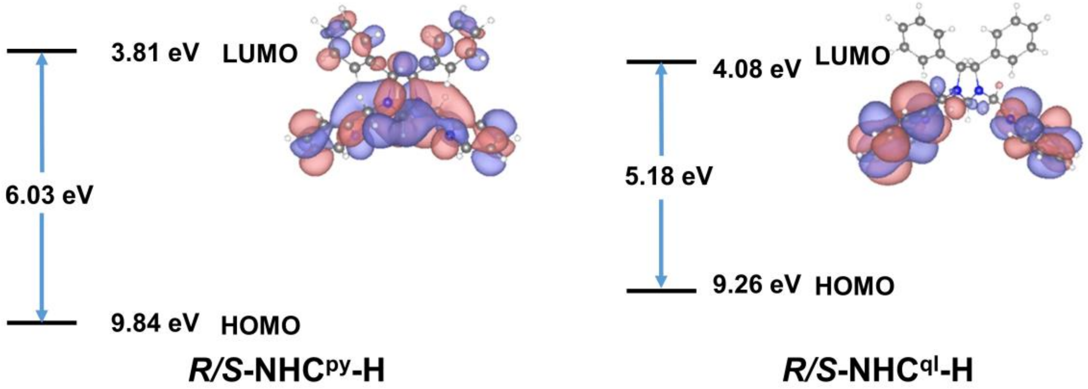

Supplementary Figure 31. HOMO−LUMO gap of *R/S*-NHC^{py}-H and *R/S*-NHC^{ql}-H.

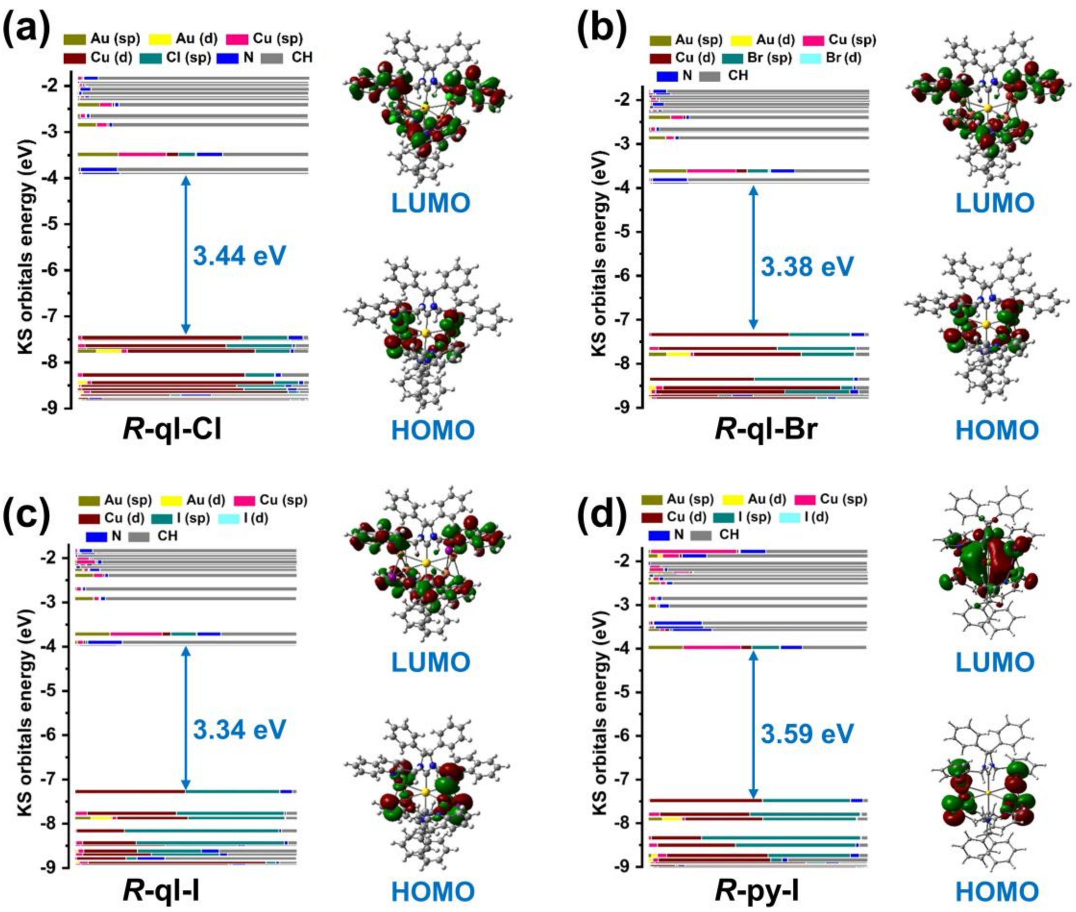

Supplementary Figure 32. Energy alignment of the MOs of comparison of KS orbital energy levels of *R*-ql-Cl (a), *R*-ql-Br (b), *R*-ql-I (c) and *R*-py-I (d). Each KS orbital was presented to indicate the relative contributions of the atomic orbitals (line length with colour labels). Visualization and of HOMO and LUMO orbitals for *R*-ql-X (X = Cl, Br, and I) and *R*-py-I in optimized structures of S_0.

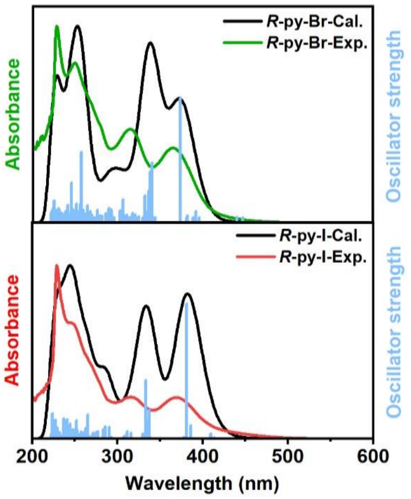

Supplementary Figure 33. The experimental optical absorption spectrum of *R*-py-Br (green), *R*-py-I (red) in CH_2Cl_2 (1 × 10^{-5} mol/L) compared to the calculated spectrum (black). Light blue bars show the individual transitions (delta function like peaks showing the relative oscillator strengths).

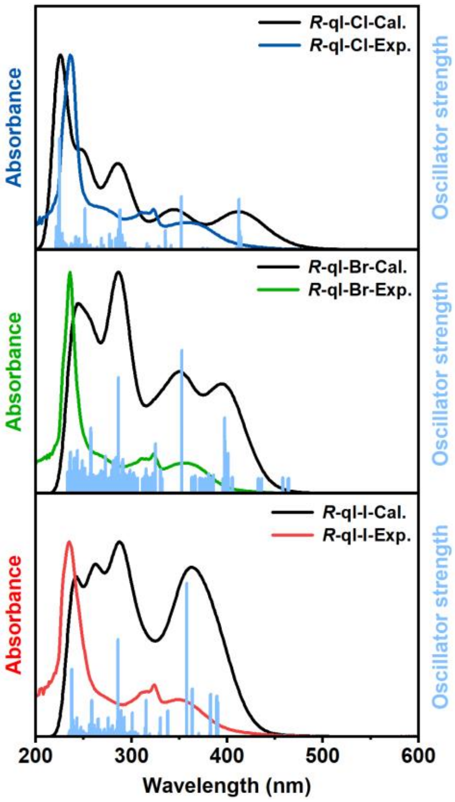

Supplementary Figure 34. The experimental optical absorption spectrum of *R*-ql-Cl (blue), *R*-ql-Br (green), *R*-ql-I (red) in CH_2Cl_2 (1 × 10^{-5} mol/L) compared to the calculated spectrum (black). Light blue bars show the individual transitions (delta function like peaks showing the relative oscillator strengths).

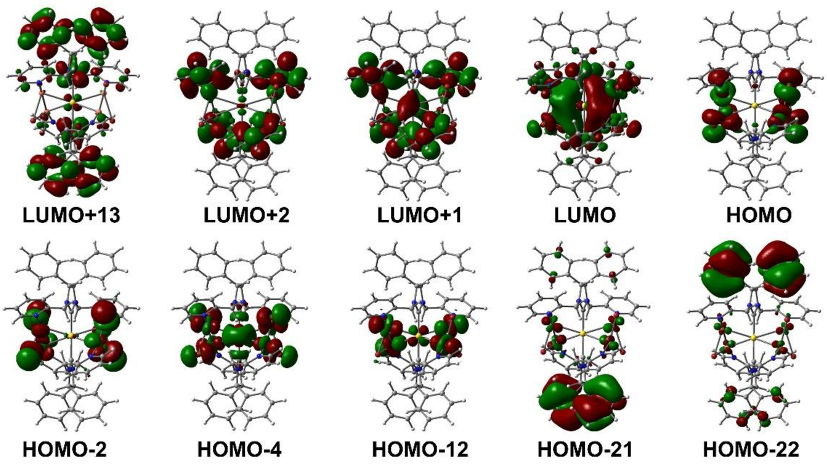

Supplementary Figure 35. Transition-involved molecular orbitals of *R*-py-Br.

Supplementary Table 13. Transition energy, oscillator strength, and orbital contributions of the strongest electronic excitations of *R*-py-Br.

Wavelength (nm)	Oscillator Strength (length representation)	Major orbital contributions (only those ≥ 10% are shown here)
373.5113	0.2269	H-4->LUMO (80%), HOMO-2->L+1 (11%)
340.2303	0.1042	H-4->L+1 (73%), H-3->L+2 (10%),
338.0964	0.0875	H-3->L+4 (30%), H-2->L+2 (60%)
337.5993	0.0548	H-3->L+2 (17%), H-1->L+2 (30%), HOMO->L+4 (33%)
336.144	0.0503	H-4->L+1 (17%), H-3->L+2 (24%), H-2->L+4 (17%), HOMO->L+4 (21%)
306.0863	0.0344	H-14->LUMO (26%), H-10->LUMO (49%)
275.9294	0.0144	H-4->L+8 (77%)
257.0562	0.1236	H-21->LUMO (64%)
245.4192	0.0659	H-22->LUMO (52%)

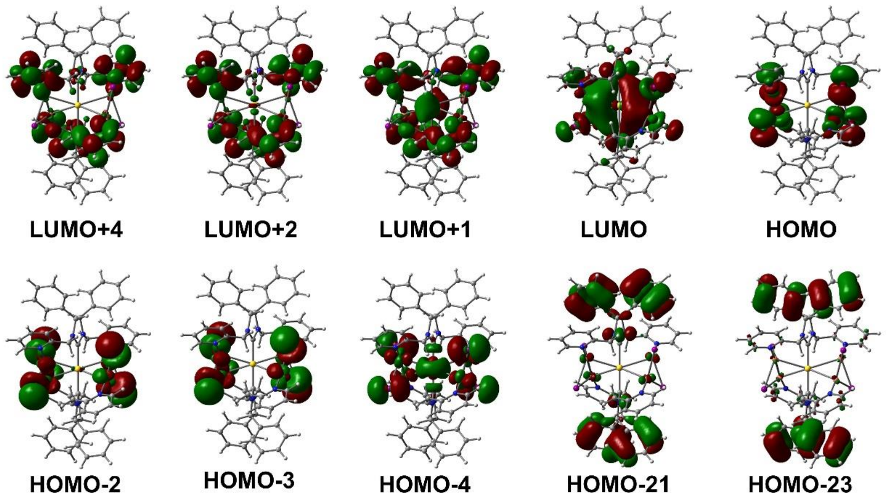

Supplementary Figure 36. Transition-involved molecular orbitals of *R*-py-I.

Supplementary Table 14. Transition energy, oscillator strength, and orbital contributions of the strongest electronic excitations of *R*-py-I.

Wavelength (nm)	Oscillator Strength (length representation)	Major orbital contributions (only those ≥ 10% are shown here)
386.3971	0.0313	H-1->L+3 (24%), HOMO->L+1 (62%)
381.4285	0.3124	H-4->LUMO (81%)
337.3696	0.0665	H-6->LUMO (82%)
333.3154	0.1356	H-3->L+2 (40%), H-2->L+4 (29%)
283.7537	0.0226	H-8->L+1 (14%), H-3->L+7 (25%), H-2->L+8 (29%)
248.5483	0.0216	H-1->L+14 (21%), H-1->L+19 (19%), HOMO->L+15 (23%)
241.2127	0.0449	H-4->L+14 (31%), H-4->L+19 (11%)

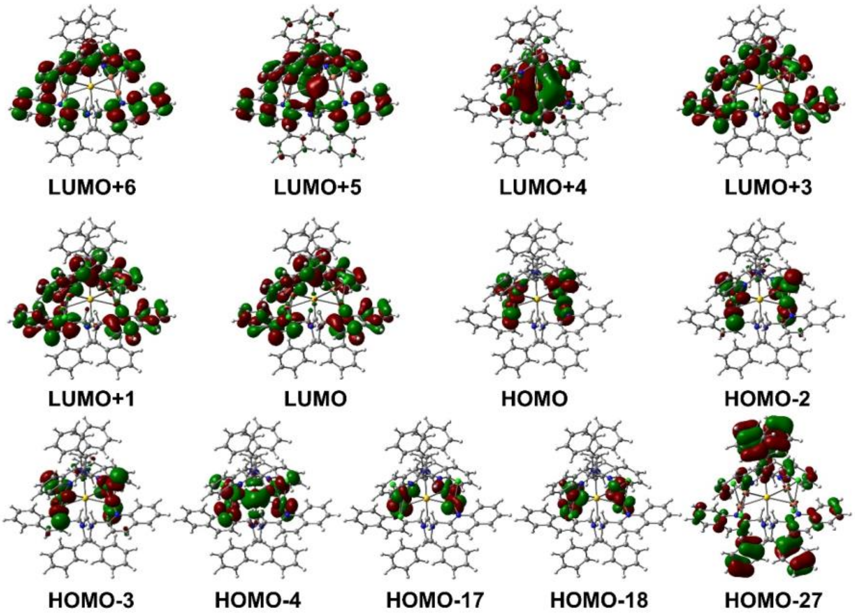

Supplementary Figure 37. Transition-involved molecular orbitals of *R*-ql-Cl.

Supplementary Table 15. Transition energy, oscillator strength, and orbital contributions of the strongest electronic excitations of *R*-ql-Cl.

Wavelength (nm)	Oscillator Strength (length representation)	Major orbital contributions (only those ≥ 10% are shown here)
412.4117	0.1669	H-3->LUMO (50%), H-2->L+3 (40%),
352.1654	0.1755	H-4->L+4 (68%)
288.0453	0.1308	H-20->L+3 (12%), H-19->L+1 (14%)
251.6508	0.136	H-29->L+4 (15%), H-23->L+4 (35%),
224.4692	0.1376	H-23->L+5 (18%), H-17->L+7 (11%), H-14->L+18 (16%)
224.2784	0.108	H-40->L+3 (40%), H-21->L+5 (10%)
224.1486	0.1459	H-19->L+5 (29%)

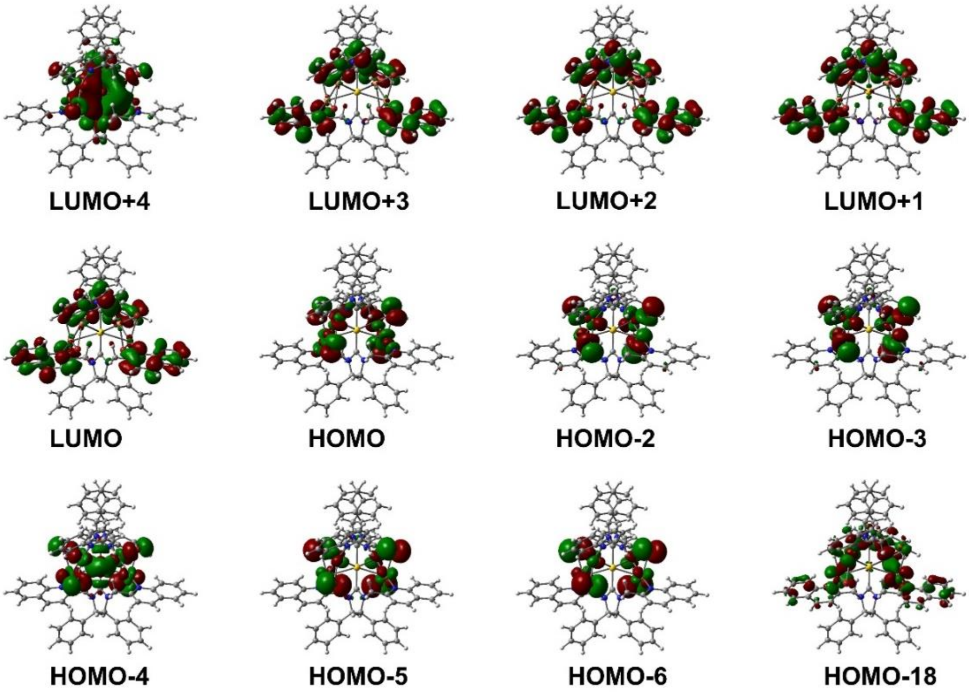

Supplementary Figure 38. Transition-involved molecular orbitals of *R*-ql-Br.

Supplementary Table 16. Transition energy, oscillator strength, and orbital contributions of the strongest electronic excitations of *R*-ql-Br.

Wavelength (nm)	Oscillator Strength (length representation)	Major orbital contributions (only those ≥ 10% are shown here)
397.8671	0.0738	H-3->L+3 (34%), H-2->L+1 (58%)
397.3698	0.1197	H-3->L+1 (49%), H-2->L+3 (42%)
352.606	0.2528	H-4->L+4 (81%)
325.1169	0.0692	H-6->LUMO (47%), H-5->L+2 (30%)
286.4547	0.1994	H-22->LUMO (14%), H-21->L+3 (16%), H-19->LUMO (18%)
257.767	0.0906	H-24->L+4 (44%), H-14->L+4 (26%)
257.6491	0.0997	H-28->L+4 (20%), H-22->L+4 (55%)

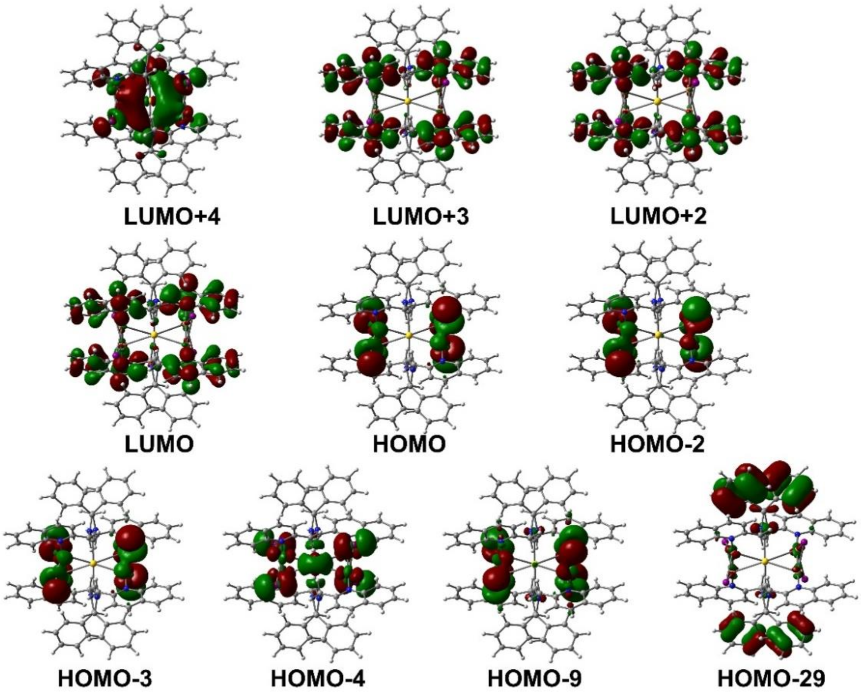

Supplementary Figure 39. Transition-involved molecular orbitals of *R*-ql-I.

Supplementary Table 17. Transition energy, oscillator strength, and orbital contributions of the strongest electronic excitations of *R*-ql-I.

Wavelength (nm)	Oscillator Strength (length representation)	Major orbital contributions (only those ≥ 10% are shown here)
389.236	0.0673	H-3->L+3 (38%), H-3->L+4 (26%), H-2->L+1 (33%)
382.7237	0.07	H-3->L+1 (32%), H-2->L+4 (52%)
363.6302	0.0789	H-4->L+3 (83%)
357.6408	0.2525	H-4->L+3 (10%), H-4->L+4 (75%)
315.399	0.0603	H-9->LUMO (32%), H-8->L+2 (28%)
285.8801	0.16	H-21->LUMO (14%), H-2->L+5 (27%)
237.5979	0.1112	H-9->L+8 (18%), H-8->L+7 (28%)

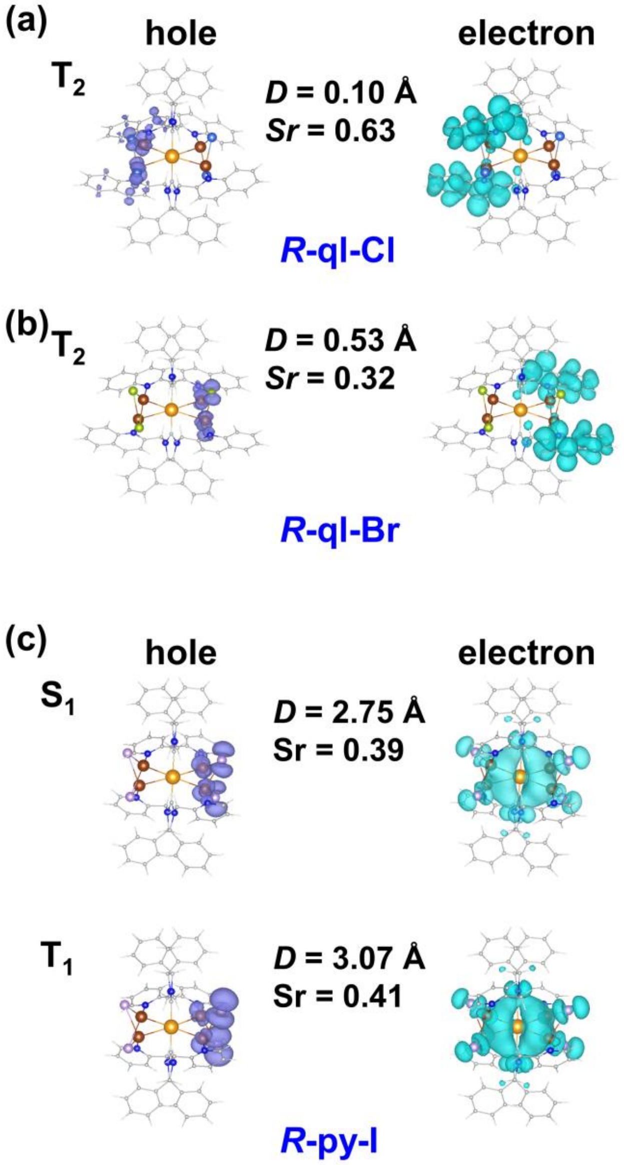

Supplementary Figure 40. The hole and electron pairs for T_2 transitions were obtained by the natural transition orbital (NTO) analysis at optimized T_2 geometries of *R*-ql-Cl (a) and *R*-ql-Br (b) (isovalue 0.02). (c) The hole and electron pairs for S_1 and T_1 transitions were obtained by the NTO analysis at optimized T_2 geometries of *R*-py-I (isovalue 0.02).

For *R*-py-I, the NHC^{py} ligands rendered the resulting *R*-py-I complexes had CT excited states T_1, and more local excitation (^{1}LE) character in S_1. At the same time, the atomic orbitals contribution of S_1 and T_1 had big differences (Supplementary Table 25). According to El-Sayed rules, *R*-py-I will have a large $\langle S_1|H_{SOC}|T_1\rangle$ value.

Supplementary Table 18. Calculated basis function contribution to hole and electron of S_1 and T_2 for the S_1, T_2 geometries of *R*-ql-Cl, respectively.

	Atom	Shell	Type	Hole	Atom	Shell	Type	Electron
S_1	2(Cu)	21	d_{xz}	34.69%	28(C)	186	p_x	9.36%
	46(Cu)	299	d_{xz}	22.37%	9(N)	72	p_x	7.71%
	2(Cu)	22	d_{xz}	4.80%	28(C)	187	p_x	7.27%
	47(Cl)	308	p_z	3.08%	16(C)	114	p_x	6.70%
	5(Cl)	49	p_x	2.98%	9(N)	73	p_x	4.36%
	5(Cl)	49	p_z	2.63%	28(C)	186	p_z	3.97%
	2(Cu)	21	d_{z^2}	2.62%	9(N)	72	p_z	3.94%
	46(Cu)	300	d_{xz}	2.57%	16(C)	115	p_x	3.65%
	47(Cl)	308	p_x	2.52%	43(C)	276	p_x	3.20%
	47(Cl)	307	p_z	2.23%	22(C)	150	p_x	3.15%
T_2	3(Cu)	32	d_{z^2}	16.28%	79(C)	506	p_x	4.78%
	48(Cu)	318	d_{z^2}	16.17%	42(C)	270	p_x	4.74%
	3(Cu)	32	$d_{x^2-y^2}$	3.74%	6(N)	54	p_x	4.06%
	48(Cu)	318	$d_{x^2-y^2}$	3.72%	53(N)	350	p_x	4.05%
	4(Cl)	41	p_x	2.88%	79(C)	507	p_x	3.71%
	49(Cl)	327	p_x	2.87%	42(C)	271	p_x	3.67%
	3(Cu)	33	d_{z^2}	2.54%	26(C)	174	p_x	3.32%
	48(Cu)	319	d_{z^2}	2.52%	61(C)	398	p_x	3.28%
	4(Cl)	40	p_x	2.09%	6(N)	55	p_x	2.26%
	49(Cl)	326	p_x	2.09%	53(N)	351	p_x	2.26%

Supplementary Table 19. Calculated basis function contribution to hole and electron of S_1 and T_2 for the S_1, T_2 geometries of *R*-ql-Br.

	Atom	Shell	Type	Hole	Atom	Shell	Type	Electron
S_1	4(Cu)	45	d_{xz}	32.99%	55(C)	299	p_x	9.49%
	75(Cu)	402	d_{xz}	21.00%	6(N)	62	p_x	7.72%
	4(Cu)	46	d_{xz}	4.32%	55(C)	300	p_x	7.36%
	76(Br)	413	p_x	3.80%	29(C)	176	p_x	6.78%
	3(Br)	33	p_z	3.48%	6(N)	63	p_x	4.38%
	3(Br)	33	p_x	3.41%	55(C)	299	p_z	4.06%
	76(Br)	412	p_x	3.19%	6(N)	62	p_z	4.01%
	3(Br)	32	p_x	3.01%	29(C)	177	p_x	3.69%
	3(Br)	32	p_z	2.94%	63(C)	335	p_x	3.20%
	76(Br)	413	p_z	2.93%	40(C)	230	p_x	3.14%
T_2	4(Cu)	45	d_{xy}	26.36%	55(C)	299	p_y	3.71%
	75(Cu)	402	d_{xy}	26.36%	113(C)	618	p_y	3.71%
	3(Br)	33	p_y	5.56%	55(C)	299	p_x	3.15%
	76(Br)	413	p_y	5.56%	113(C)	618	p_x	3.15%
	3(Br)	32	p_y	4.79%	6(N)	62	p_y	3.08%
	76(Br)	412	p_y	4.79%	81(N)	450	p_y	3.08%
	4(Cu)	46	d_{xy}	3.19%	55(C)	300	p_y	2.92%
	75(Cu)	403	d_{xy}	3.19%	113(C)	619	p_y	2.92%
	6(N)	62	p_y	1.39%	6(N)	62	p_x	2.91%
	81(N)	450	p_y	1.39%	81(N)	450	p_x	2.91%

Supplementary Table 20. Calculated basis function contribution to hole and electron of S_1 and T_1 for the S_1, T_1 geometries of *R*-ql-I.

	Atom	Shell	Type	Hole	Atom	Shell	Type	Electron
S_1	9(Cu)	94	d_{xz}	28.22%	63(C)	358	p_x	9.40%
	8(Cu)	83	d_{xz}	17.98%	14(N)	124	p_x	7.48%
	5(I)	50	p_x	5.20%	63(C)	359	p_x	7.31%
	4(I)	39	p_x	5.14%	80(C)	439	p_x	6.62%
	5(I)	49	p_x	5.06%	14(N)	125	p_x	4.26%
	4(I)	40	p_x	5.02%	63(C)	358	p_z	4.18%
	4(I)	40	p_z	4.02%	14(N)	124	p_z	4.06%
	4(I)	39	p_z	3.94%	80(C)	440	p_x	3.58%
	5(I)	49	pz	3.42%	102(C)	544	p_x	3.21%
	9(Cu)	95	d_{xz}	3.42%	78(C)	430	p_x	3.16%
T_1	7(Cu)	72	d_{z^2}	9.22%	134(C)	691	p_x	10.81%
	38(C)	235	p_x	7.59%	134(C)	692	p_x	8.22%
	95(C)	511	p_x	6.81%	16(N)	136	p_x	6.94%
	16(N)	136	p_x	6.50%	72(C)	400	p_x	5.98%
	134(C)	691	p_x	6.03%	95(C)	511	p_x	5.61%
	38(C)	236	p_x	5.41%	38(C)	235	p_x	5.00%
	95(C)	512	p_x	4.54%	95(C)	512	p_x	4.05%
	134(C)	692	p_x	3.91%	38(C)	236	p_x	4.00%
	16(N)	137	p_x	3.74%	16(N)	137	p_x	3.92%
	84(C)	460	p_x	2.99%	89(C)	484	p_x	3.20%

Supplementary Table 21. Computed SOCME values of *R*-ql-X (X = Cl, Br, and I) nanoclusters at their S_1 optimized structures.

	$\langle S_1 \vert H_{SOC} \vert T_1 \rangle$	$\langle S_1 \vert H_{SOC} \vert T_2 \rangle$	$\langle S_1 \vert H_{SOC} \vert T_3 \rangle$	$\langle S_1 \vert H_{SOC} \vert T_4 \rangle$	$\langle S_1 \vert H_{SOC} \vert T_5 \rangle$
***R*-ql-Cl**	45.25	271.34	3.2101	0.7559	0.4555
***R*-ql-Br**	14.05	95.92	19.20	1.57	1.59
***R*-ql-I**	70.07	234.26	37.88	5.64	1.35
***R*-py-I**	96.03	505.05	870.85	827.03	198.15

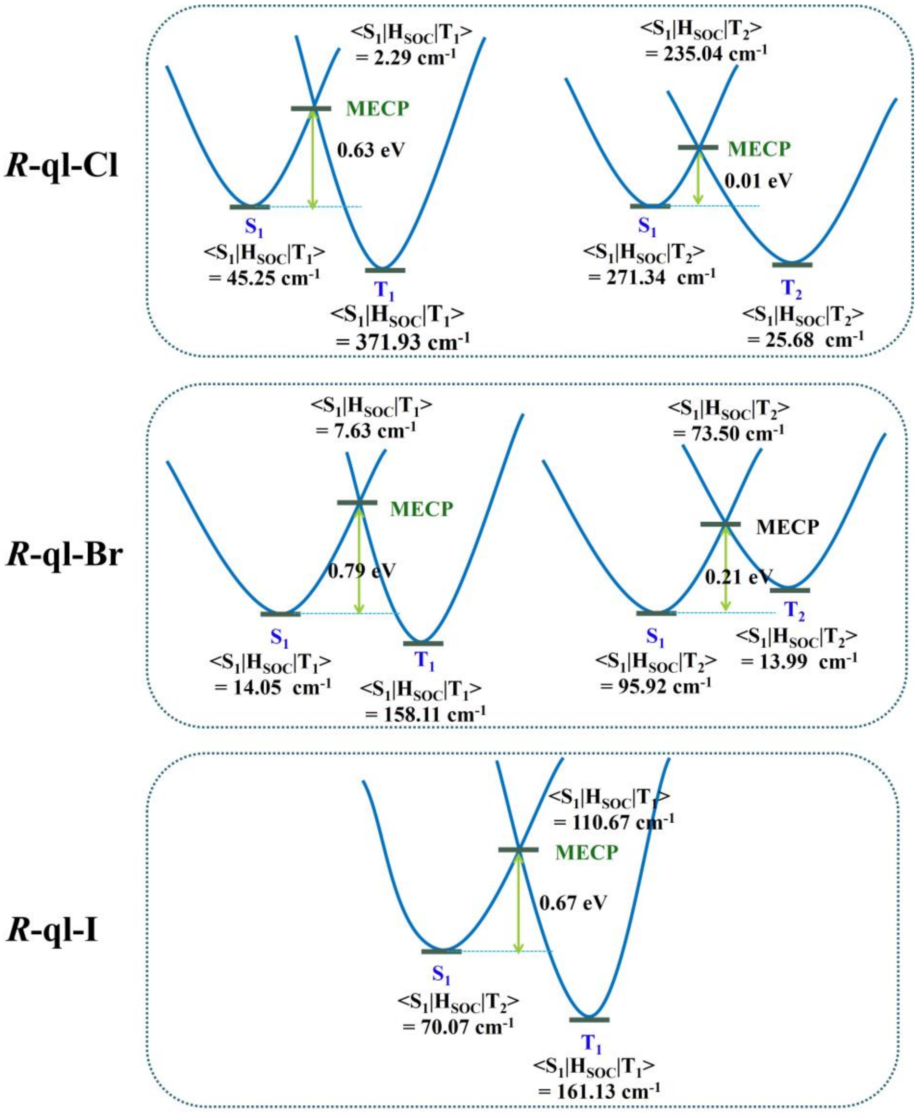

Supplementary Figure 41. Calculated spin-orbit coupling matrix elements (SOCME) values for the geometries of S_1, T_1/T_2 and minimum energy crossing point (MECP) between S_1 and T_1/T_2 for *R*-ql-Cl, *R*-ql-Br, and *R*-ql-I.

Supplementary Table 22. Calculated basis function contribution to hole and electron of S_1 and T_2 for the MECP between S_1 and T_2 geometries of *R*-ql-Cl.

	Atom	Shell	Type	Hole	Atom	Shell	Type	Electron
S_1	2(Cu)	21	d_{xz}	35.08%	28(C)	186	p_x	9.41%
	46(Cu)	299	d_{xz}	22.07%	9(N)	72	p_x	7.72%
	2(Cu)	22	d_{xz}	4.87%	28(C)	187	p_x	7.32%
	47(Cl)	308	p_z	3.06%	16(C)	114	p_x	6.67%
	5(Cl)	49	p_x	2.98%	9(N)	73	p_x	4.36%
	2(Cu)	21	d_{z^2}	2.69%	28(C)	186	p_z	4.00%
	5(Cl)	49	p_z	2.57%	9(N)	72	p_z	3.97%
	47(Cl)	308	p_x	2.55%	16(C)	115	p_x	3.63%
	46(Cu)	300	d_{xz}	2.53%	43(C)	276	p_x	3.19%
	47(Cl)	307	p_z	2.22%	22(C)	150	p_x	3.12%
T_2	2(Cu)	21	d_{z^2}	18.76%	28(C)	186	p_x	9.45%
	2(Cu)	21	$d_{x^2-y^2}$	6.11%	28(C)	187	p_x	7.32%
	28(C)	186	p_x	4.50%	9(N)	72	p_x	7.30%
	22(C)	150	p_x	3.31%	16(C)	114	p_x	6.13%
	43(C)	276	p_x	3.21%	9(N)	73	p_x	4.09%
	9(N)	72	p_x	3.12%	28(C)	186	p_z	4.01%
	2(Cu)	22	d_{z^2}	3.09%	9(N)	72	p_z	3.69%
	28(C)	187	p_x	3.02%	43(C)	276	p_x	3.67%
	2(Cu)	21	d_{xz}	2.78%	22(C)	150	p_x	3.37%
	22(C)	151	p_x	2.61%	16(C)	115	p_x	3.29%

Supplementary Table 23. Calculated basis function contribution to hole and electron of S_1 and T_2 for the MECP between S_1 and T_2 of *R*-ql-Br geometries.

	Atom	Shell	Type	Hole	Atom	Shell	Type	Electron
S_1	4(Cu)	45	d_{xy}	26.81%	55(C)	299	p_y	4.52%
	75(Cu)	402	d_{xy}	26.81%	113(C)	618	p_y	4.52%
	3(Br)	33	p_x	4.91%	29(C)	176	p_y	4.14%
	76(Br)	413	p_x	4.91%	89(C)	498	p_y	4.14%
	3(Br)	32	p_x	4.16%	6(N)	62	p_y	3.83%
	76(Br)	412	p_x	4.16%	81(N)	450	p_y	3.83%
	4(Cu)	46	d_{xy}	3.20%	55(C)	300	p_y	3.53%
	75(Cu)	403	d_{xy}	3.20%	113(C)	619	p_y	3.53%
	3(Br)	33	p_y	2.15%	1(Au)	9	p_x	2.49%
	76(Br)	413	p_y	2.15%	29(C)	177	p_y	2.49%
T_2	4(Cu)	45	d_{xy}	26.20%	55(C)	299	p_y	4.92%
	75(Cu)	402	d_{xy}	26.20%	113(C)	618	p_y	4.92%
	3(Br)	33	p_x	4.68%	29(C)	176	p_y	4.30%
	76(Br)	413	p_x	4.68%	89(C)	498	p_y	4.30%
	3(Br)	32	p_x	3.99%	6(N)	62	p_y	4.16%
	76(Br)	412	p_x	3.99%	81(N)	450	p_y	4.16%
	4(Cu)	46	d_{xy}	3.09%	55(C)	300	p_y	3.82%
	75(Cu)	403	d_{xy}	3.09%	113(C)	619	p_y	3.82%
	3(Br)	33	p_y	2.17%	29(C)	177	p_y	2.46%
	76(Br)	413	p_y	2.17%	89(C)	499	p_y	2.46%

Supplementary Table 24. Calculated basis function contribution to hole and electron of S_1 and T_1 for the MECP between S_1 and T_1 of *R*-ql-I geometries.

	Atom	Shell	Type	Hole	Atom	Shell	Type	Electron
S_1	8(Cu)	83	d_{xz}	22.39%	63(C)	358	p_x	8.97%
	9(Cu)	94	d_{xz}	17.12%	63(C)	359	p_x	6.77%
	5(I)	50	p_x	6.40%	14(N)	124	p_x	5.69%
	5(I)	49	p_x	6.02%	80(C)	439	p_x	4.74%
	4(I)	40	p_z	5.72%	63(C)	358	p_z	3.79%
	4(I)	39	p_z	5.49%	78(C)	430	p_x	3.65%
	4(I)	39	p_x	3.43%	102(C)	544	p_x	3.63%
	4(I)	40	p_x	3.16%	14(N)	124	p_z	3.59%
	9(Cu)	95	d_{xz}	2.25%	14(N)	125	p_x	3.29%
	8(Cu)	84	d_{xz}	2.24%	80(C)	439	p_z	2.87%
T_1	8(Cu)	83	d_{xz}	21.63%	63(C)	358	p_x	8.97%
	9(Cu)	94	d_{xz}	18.14%	63(C)	359	p_x	6.75%
	5(I)	50	p_x	6.27%	14(N)	124	p_x	5.74%
	5(I)	49	p_x	5.90%	80(C)	439	p_x	4.81%
	4(I)	40	p_z	5.62%	63(C)	358	p_z	3.80%
	4(I)	39	p_z	5.40%	14(N)	124	p_z	3.65%
	4(I)	39	p_x	3.47%	78(C)	430	p_x	3.58%
	4(I)	40	p_x	3.17%	102(C)	544	p_x	3.58%
	9(Cu)	95	d_{xz}	2.41%	14(N)	125	p_x	3.34%
	8(Cu)	84	d_{xz}	2.14%	80(C)	439	p_z	2.90%

Supplementary Table 25. Calculated basis function contribution to hole and electron of S_1 and T_1 for the S_1, T_1 geometries of *R*-py-I.

	Atom	Shell	Type	Hole	Atom	Shell	Type	Electron
S_1	6(Cu)	61	d_{xz}	16.58%	1(Au)	9	p_y	26.44%
	7(Cu)	72	d_{xz}	16.58%	24(C)	185	p_y	4.12%
	2(I)	20	p_x	8.00%	26(C)	197	p_y	4.12%
	3(I)	30	p_x	8.00%	24(C)	184	p_y	3.73%
	2(I)	19	p_x	7.92%	26(C)	196	p_y	3.73%
	3(I)	29	p_x	7.92%	7(Cu)	71	p_y	2.54%
	6(Cu)	61	d_{z^2}	5.69%	6(Cu)	60	p_y	2.53%
	7(Cu)	72	d_{z^2}	5.69%	15(N)	130	p_y	2.27%
	6(Cu)	62	d_{xz}	2.09%	17(N)	142	p_y	2.27%
	7(Cu)	73	d_{xz}	2.09%	12(N)	112	p_y	2.26%
T_1	2(I)	19	p_x	10.65%	1(Au)	9	p_y	25.62%
	3(I)	29	p_x	10.64%	24(C)	185	p_y	3.91%
	2(I)	20	p_x	10.19%	26(C)	197	p_y	3.91%
	3(I)	30	p_x	10.18%	26(C)	196	p_y	3.37%
	6(Cu)	61	d_{z^2}	6.20%	24(C)	184	p_y	3.36%
	7(Cu)	72	d_{z^2}	6.19%	6(Cu)	60	p_y	2.49%
	3(I)	29	p_z	4.12%	7(Cu)	71	p_y	2.49%
	2(I)	19	p_z	4.11%	7(Cu)	67	s	2.27%
	3(I)	30	p_z	3.92%	6(Cu)	56	s	2.26%
	2(I)	20	p_z	3.91%	12(N)	112	p_y	2.26%

Supplementary Table 26. Au atom contribution to holes and electrons of S_1, T_1, T_2 states of *R*-ql-Cl, *R*-ql-Br, *R*-ql-I and *R*-py-I.

geometries	**Au atom**	**Hole**	**Electron**	**Overlap**	**Diff.**
	S_1	0.27 %	0.27 %	0.27 %	0.39 %
R-ql-Cl	T_1	0.03 %	0.29 %	0.10 %	0.26 %
	T_2	0.05 %	0.26 %	0.26 %	0.21%
	S_1	0.13 %	0.60 %	0.28 %	0.46 %
R-ql-Br	T_1	0.07 %	0.27 %	0.14 %	0.20 %
	T_2	0.08 %	0.18 %	0.12 %	0.10 %
R-ql-I	S_1	0.05 %	0.52 %	0.15 %	0.47 %
	T_1	0.16 %	0.11 %	0.13 %	-0.05 %
R-py-I	S_1	0.64 %	28.38 %	4.28 %	27.74 %
	T_1	0.37 %	27.42 %	3.17 %	27.05 %

Supplementary Table 27. Cu atom contribution to holes and electron of S_1, T_1, T_2 states of *R*-ql-Cl, *R*-ql-Br, *R*-ql-I, and *R*-py-I.

geometries	Cu atom	Hole	Electron	Overlap	Diff.
	S_1	71.02%	2.42%	11.45%	-68.61%
R-ql-Cl	T_1	47.03%	3.98%	13.66%	-43.05%
	T_2	52.85%	2.44%	11.39%	-50.40%
	S_1	65.01%	2.19%	10.34%	-62.82%
R-ql-Br	T_1	37.42%	3.06%	10.58%	-34.35%
	T_2	62.98%	1.82%	10.64%	-61.20%
R-ql-I	S_1	54.78%	1.90%	8.75%	-52.87%
	T_1	14.04%	1.65%	4.81%	-12.38%
R-py-I	S_1	57.11%	18.66%	27.48%	-38.45%
	T_1	36.19%	20.48%	21.87%	-15.71%

Supplementary Table 28. Halid X (Cl, Br, and I) ions contribution to holes and electron of S_1, T_1, T_2 states of ***R*-ql-Cl**, ***R*-ql-Br**, ***R*-ql-I,** and ***R*-py-I**.

geometries	X (Cl, Br, I)	Hole	Electron	Overlap	Diff.
	S_1	19.89%	0.53%	3.17%	-19.37%
R-ql-Cl	T_1	12.40%	0.92%	3.33%	-11.49%
	T_2	15.37%	0.49%	2.70%	-14.86%
	S_1	25.89%	0.61%	3.83%	-25.28%
R-ql-Br	T_1	12.49%	0.84%	3.20%	-11.65%
	T_2	24.58%	0.38%	2.94%	-24.22%
R-ql-I	S_1	36.28%	0.73%	4.92%	-35.55%
	T_1	5.81%	0.59%	1.82%	-5.22%
R-py-I	S_1	34.82%	17.71%	19.70%	-17.12%
	T_1	60.10%	18.04%	24.71%	-42.06%

Supplementary Table 29. Computed spin-orbit coupling matrix elements (SOCME) values, reorganization energies (λ), intersystem crossing rate (k_{ISC}) and reverse intersystem crossing rate (k_{RISC}) of *R*-ql-X (X = Cl, Br, and I) and *R*-py-I nanoclusters at their $S_1 \leftrightarrow T_n$.

Cluster	ISC/RISC	ISC		RISC		
		λ eV	k_{ISC} S^{-1}	SOCME cm^{-1}	λ eV	k_{RISC} S^{-1}
R-ql-Cl	$S_1 \leftrightarrow T_1$	2.30×10^{-3}	0	371.93	3.65×10^{-1}	5.49×10^{8}
	$S_1 \leftrightarrow T_2$	3.22×10^{-2}	7.28×10^{13}	25.68	2.39×10^{-1}	7.59×10^{9}
R-ql-Br	$S_1 \leftrightarrow T_1$	1.29×10^{-1}	1.04×10^{11}	158.11	3.08×10^{-1}	4.22×10^{9}
	$S_1 \leftrightarrow T_2$	5.39×10^{-2}	1.51×10^{12}	13.99	8.56×10^{-2}	1.49×10^{11}
R-ql-I	$S_1 \leftrightarrow T_1$	5.22×10^{-2}	2.60×10^{12}	161.10	2.54×10^{-1}	7.05×10^{10}
R-py-I	$S_1 \leftrightarrow T_1$	1.47×10^{-4}	0	13.25	2.61×10^{-2}	7.29×10^{7}

Supplementary Table 30. The spin-orbit Coupling Matrix Elements (SOCME, cm^{-1}) Values, Reorganization Energies Treated in a Classical Way (λ, eV) as well as Interna Conversion rate [k_{IC} (s^{-1})] of *R*-ql-Cl and *R*-ql-Br.

$T_2 \rightarrow T_1$	SOCME (cm^{-1})	λ (eV)	k_{IC} (s^{-1})
R-ql-Cl	46.40	0.20	1.20×10^{12}
R-ql-Br	29.68	0.21	4.73×10^{11}

Supplementary Table 31. Experimental and computed vertical emission energies (E_{em}), oscillator strengths (f_{em}), as well as experimental and calculated radiative transition rates of *R*-ql-X (X = Cl, Br, and I) and *R*-py-I from S_1 to S_0 or T_1 to S_0.

Cluster	Exp-E_{em} eV	Cal-E_{em} eV	f_{em}	Exp-$k_{r,RT}$ s^{-1}	Cal-$k_{r,RT}$ s^{-1}
[a]*R*-ql-Cl	1.975	2.059	3.7×10^{-4}	3.31×10^{4}	6.79×10^{4}
[a]*R*-ql-Br	2.068	2.056	5.2×10^{-4}	8.11×10^{4}	9.56×10^{4}
[1a]*R*-ql-I	2.024	2.100	1.03×10^{-3}	4.61×10^{5}	1.97×10^{5}
[2b]*R*-ql-I	1.771	1.713	-	-	-
[b]*R*-py-I	1.966	1.869	2.3×10^{-4}	3.00×10^{5}	3.55×10^{4}

1 and 2 represent high-energy and low-energy emissions, respectively.

a and b represent fluorescence and phosphorescence, respectively.

10.7. CD and CPL spectra

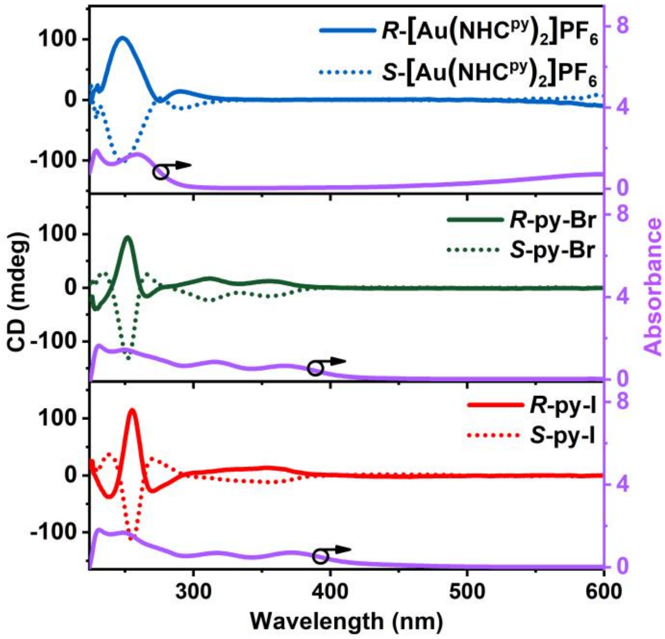

Supplementary Figure 42. CD (left) and UV (purple line; right) spectra of *R/S*-$[Au(NHC^{py})_2]PF_6$ and *R/S*-py-X (X = Br and I) in CH_2Cl_2 (1×10^{-5} mol/L) under ambient conditions.

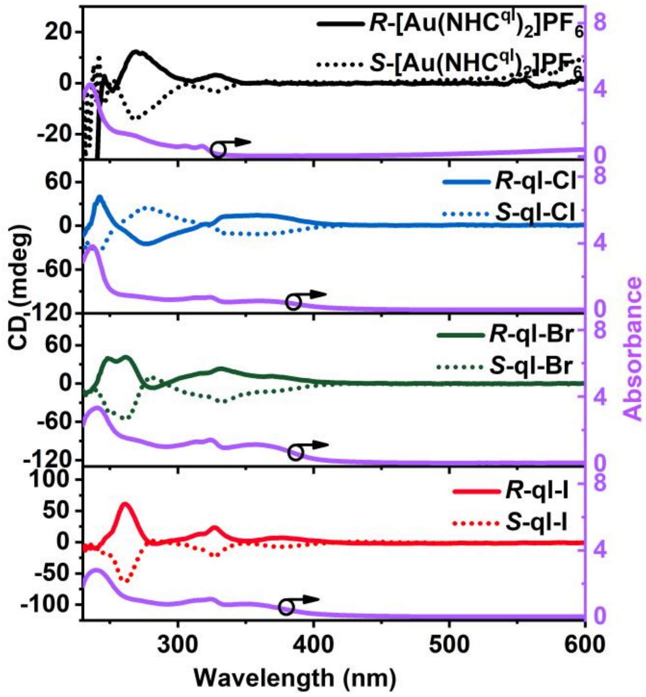

Supplementary Figure 43. CD (left) and UV (purple line; right) spectra of *R/S*-$[Au(NHC^{ql})_2]PF_6$ and *R/S*-ql-X (X = Cl, Br, and I) in CH_2Cl_2 (1×10^{-5} mol/L) under ambient conditions.

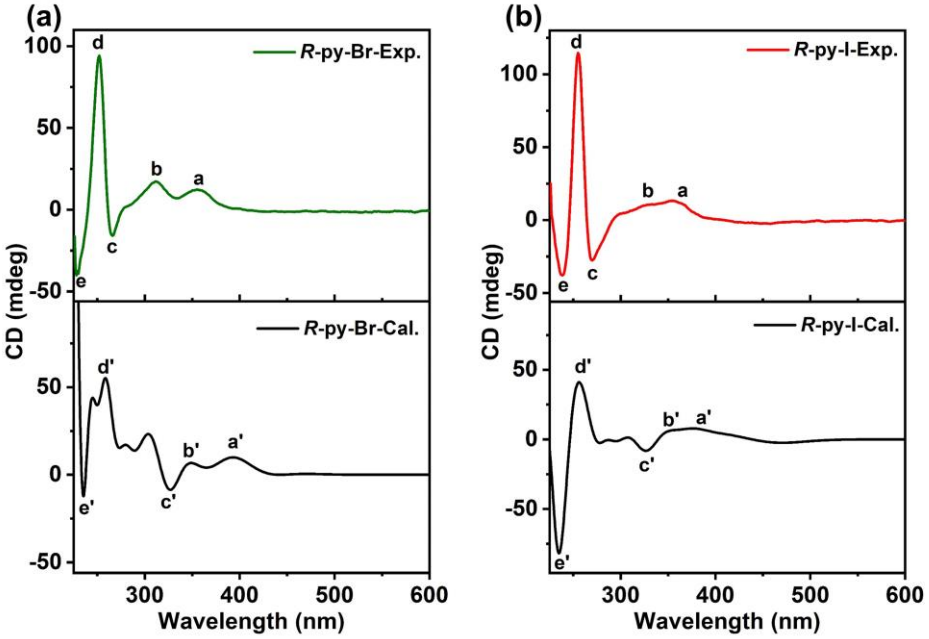

Supplementary Figure 44. Experimental CD spectra of *R*-py-Br (a) and *R*-py-I (b) in CH_2Cl_2 (1×10^{-5} mol/L) compared to the calculated spectra.

For *R*-py-Br, the signals in the low-energy regions of 340−400 nm are related to the absorption peak at 363 nm corresponding to the CD transition at 357 nm (peak a), which was ascribed to the transitions from HOMO-4 to LUMO. The UV absorption band at 313 nm corresponded to the CD transition at 311 nm (peak b), which was ascribed to the transitions from HOMO-4 to LUMO+1, and HOMO-3 to LUMO+2. And the UV absorption band centered at 271 nm was completely consistent with the CD transition at 265 nm (peak c), which was derived from HOMO-4 to LUMO+8. The UV band at 249 nm corresponded to the CD transition at 251 nm (peak d) and was mainly attributed to the HOMO-21 to LUMO transitions. The UV absorption band at 227 nm corresponded to the CD transition at 228 nm (peak e), which was ascribed to the transitions from HOMO-22 to LUMO.

For *R*-py-I, the signals in the low-energy regions of 340−400 nm are related to the absorption peak at 370 nm corresponding to the CD transition at 359 nm (peak a), which was ascribed to the transitions from HOMO-4 to LUMO. The UV absorption band at 317 nm corresponded to the CD transition at 326 nm (peak b), which was ascribed to the transitions from HOMO-3 to LUMO+2, and HOMO-2 to LUMO+4. And the UV

absorption band centered at 271 nm ascribed to the CD transition at 269 nm (peak c), which was derived from HOMO-3 to LUMO+7, and HOMO-2 to LUMO+8. The UV band at 250 nm corresponded to the CD transition at 255 nm (peak d) and was mainly attributed to the HOMO-1 to LUMO+14, LUMO+19, and HOMO to LUMO+15 transitions. The UV absorption band at 229 nm corresponded to the CD transition at 234 nm (peak e), which was ascribed to the transitions from HOMO-4 to LUMO+14 and LUMO+19.

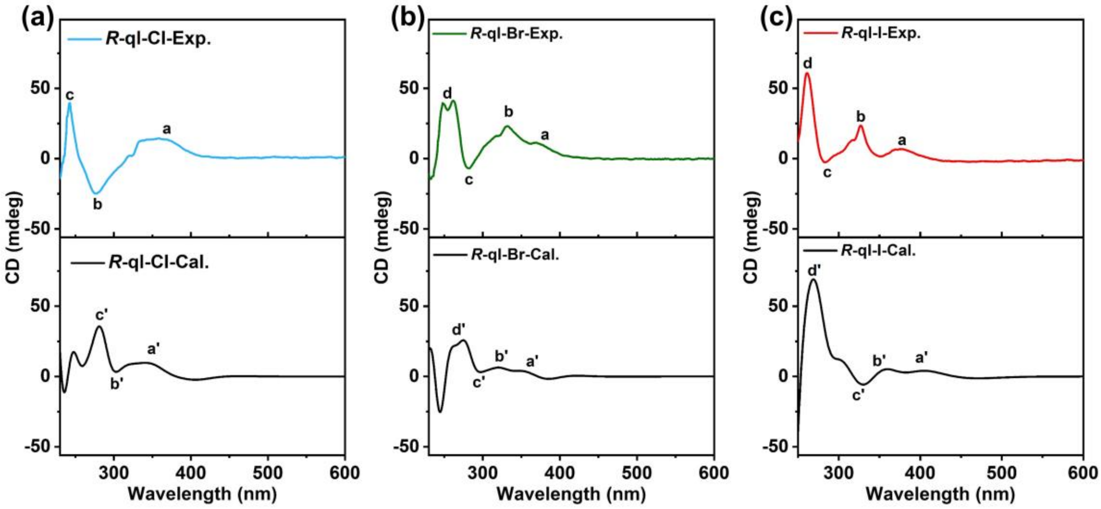

Supplementary Figure 45. Experimental CD spectra of *R*-ql-Cl (a), *R*-ql-Br (b), and *R*-ql-I (c) in CH_2Cl_2 (1×10^{-5} mol/L) compared to the calculated spectra.

For *R*-ql-Cl, the signals in the low-energy regions of 320−400 nm are related to the absorption peak at 362 nm corresponding to the CD transition at 366 nm (peak a), which was ascribed to the transitions from HOMO-4 to LUMO+4. The UV−Vis absorption band at 270 nm corresponded to the CD transition at 275 nm (peak b), which was ascribed to the transitions from HOMO-20 to LUMO+3, and HOMO-19 to LUMO+1. The UV band at 235 nm corresponded to the CD transition at 242 nm (peak c) and was mainly attributed to the HOMO-29 to LUMO+4, and HOMO-23 to LUMO+4 transitions.

For *R*-ql-Br, the signals in the low-energy regions of 340−400 nm are related to the absorption peak at 357 nm corresponding to the CD transition at 371 nm (peak a), which was ascribed to the transitions from HOMO-4 to LUMO +4. The UV absorption band at 323 nm corresponded to the CD transition at 332 nm (peak b), which was ascribed to the transitions from HOMO-6 to LUMO, and HOMO-5 to LUMO+2. And the UV absorption band centered at 270 nm ascribed to the CD transition at 280 nm (peak c), which was derived from HOMO-22 to LUMO, HOMO-21 to LUMO+3, and HOMO-19 to LUMO. The UV band at 235 nm corresponded to the CD transition at 260 nm (peak d) and was mainly attributed to the HOMO-24, HOMO-14 to LUMO+4 transitions.

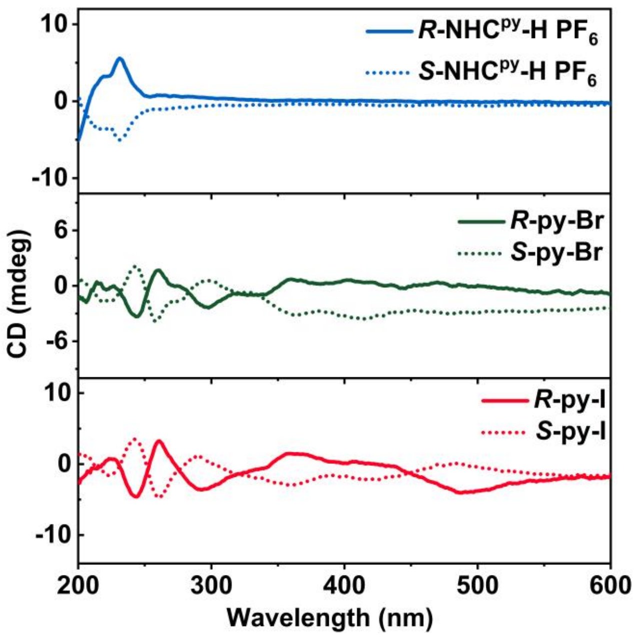

Supplementary Figure 46. CD spectra of ligand *R/S*-NHC^{py}-H PF_6 and cluser *R/S*-py-X (X = Br and I) in the solid state

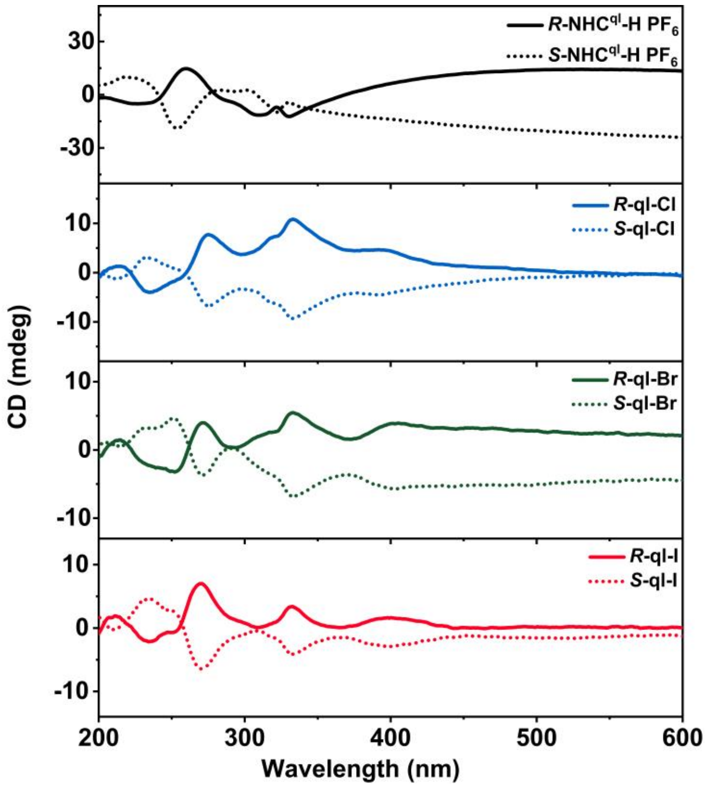

Supplementary Figure 47. CD spectra of ligand *R/S*-NHC^{ql}-H PF_6 and *R/S*-ql-X (X = Cl, Br, and I) cluster in the solid state.

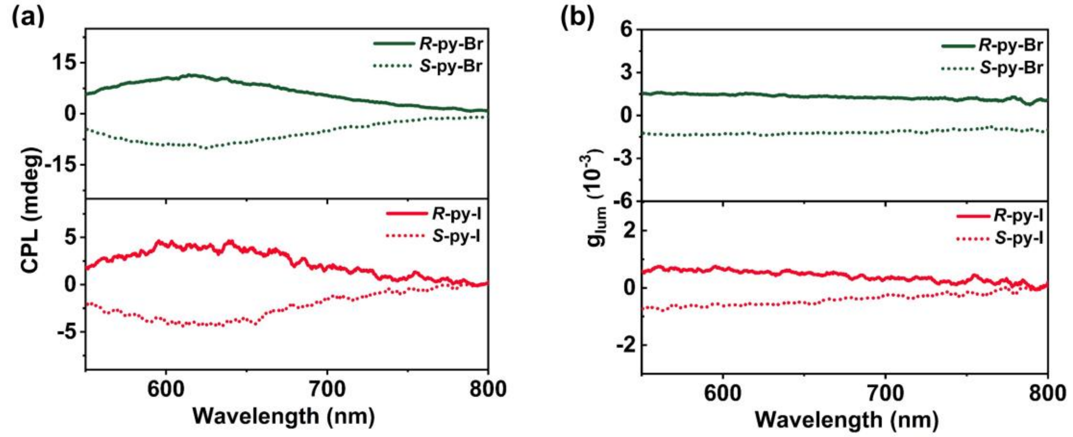

Supplementary Figure 48. CPL spectra (a) and corresponding g_{lum} values (b) of *R/S*-py-X (X = Br and I) in the solid state.

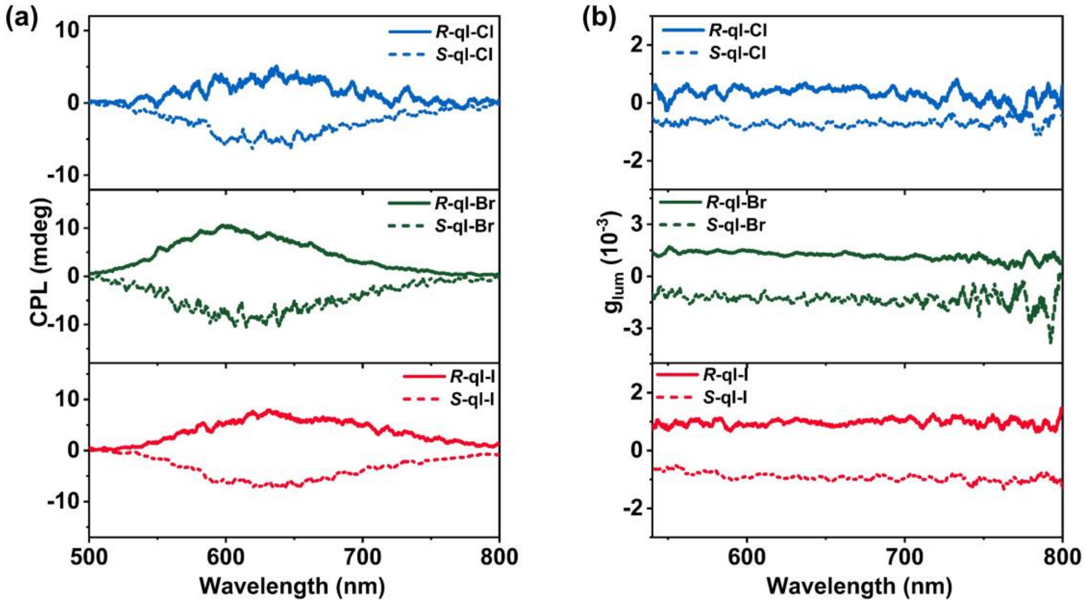

Supplementary Figure 49. CPL spectra (a) and corresponding g_{lum} values (b) of *R/S*-ql-X (X = Cl, Br, and I) in the solid state.

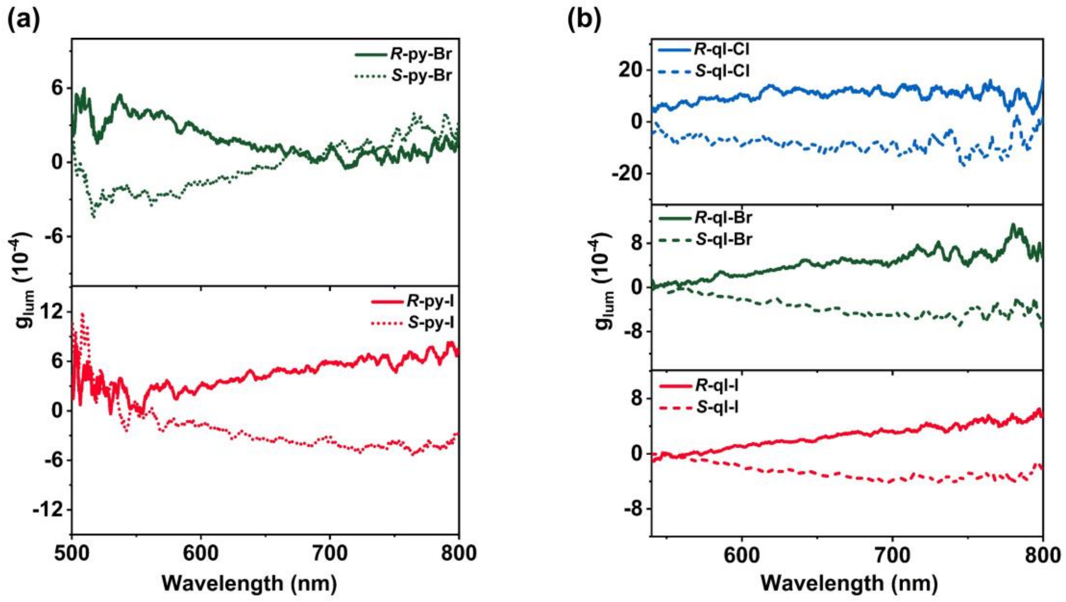

Supplementary Figure 50. g_{lum} values *R/S*-py-X (X = Br and I) (a) of *R/S*-ql-X (X = Cl, Br, and I) (b) in CH_2Cl_2 (1×10^{-5} mol/L) under ambient conditions.

Supplementary Table 32. Experimental g_{lum} values of *R*-ql-X (X = Cl, Br, and I) and *R*-py-X (X = Br and I) in the solid and solution states; Theoretical g_{lum} values and rotatory strength of *R*-ql-X (X = Cl, Br, and I) and *R*-py-X (X = Br and I).

Cluster	Exp-Solid ($\times 10^{-3}$)	Exp-Solution ($\times 10^{-3}$)	Calculated ($\times 10^{-3}$)	Rotatory strength (10^{-40} esu cm erg G^{-1})
***R*-py-Br**	1.5	0.4	7.3	0.87
***R*-py-I**	0.6	0.6	1.4	8.36
***R*-ql-Cl**	0.7	0.9	8.3	1.02
***R*-ql-Br**	1.5	0.5	2.8	0.49
***R*-ql-I**	1.1	0.4	3.4	0.41

10.8. OLED

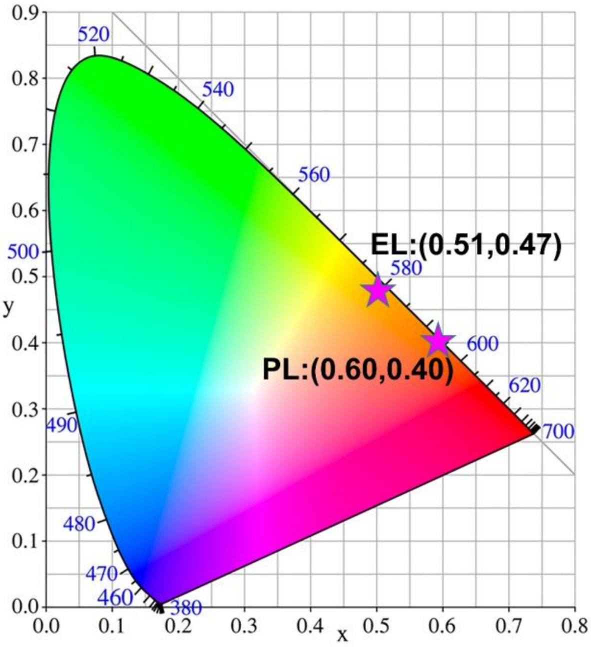

Supplementary Figure 51. The CIE coordinates of EL and PL of OLED devices.

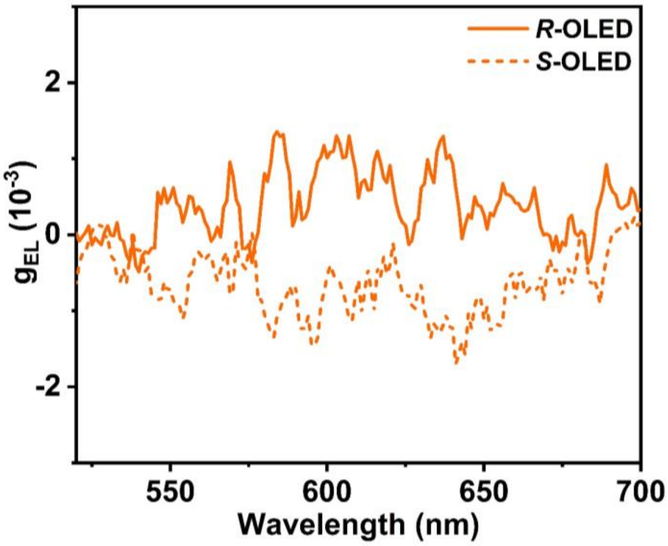

Supplementary Figure 52. Corresponding g_{lum} values of CP-OLED devices.

Supplementary References

1. Hooper, T. N. et al. synthesis, structure and reactivity of stable homoleptic gold(I) alkene cations. *Chem. Eur. J.* **15**, 12196-12200 (2009).
2. Magill, A. M. et al. Palladium(II) complexes containing mono-, bi- and tridentate carbene ligands. Synthesis, characterisation and application as catalysts in c-c coupling reactions. *J. Organomet. Chem.* **617-618**, 546-560 (2001).
3. He, W., Ge, Y.-C. & Tan, C.-H. Halogen-bonding-induced hydrogen transfer to C=N bond with hantzsch ester. *Org. Lett.* **16**, 3244-3247 (2014).
4. Dolomanov, O. V., Bourhis, L. J., Gildea, R. J., Howard, J. A. K. & Puschmann, H. *OLEX2*:A complete structure solution, refinement and analysis program. *J. Appl. Cryst.* **42**, 339-341 (2009).
5. Gaussian 16, Revision C.01, Frisch, M. J. et al. Gaussian, Inc., Wallingford CT, (2016).
6. Lu, T. & Chen, F. Multiwfn: a multifunctional wavefunction analyzer. *J. Comput. Chem.* **33**, 580-592 (2012).
7. Liu, Z., Lu, T. & Chen, Q. An Sp-hybridized all-carboatomic ring, cyclo[18]carbon: electronic structure, electronic spectrum, and optical nonlinearity. *Carbon* **165**, 461-467 (2020).
8. Perdew, J. P. Density-functional approximation for the correlation energy of the inhomogeneous electron gas. *Phys. Rev. B* **33**, 8822-8824 (1986).
9. Marcus, R. A. Electron transfer reactions in chemistry. Theory and experiment. *Rev. Mod. Phys*. **65**, 599-610 (1993).
10. Beljonne, D., Shuai, Z., Pourtois, G. & Bredas, J. L. Spin-orbit coupling and intersystem crossing in conjugated polymers: a configuration interaction description. *J. Phys. Chem. A* **105**, 3899-3907 (2001).
11. Kohler, A. & Bassler, H. Triplet states in organic semi-conductors. *Mater. Sci. Eng. R* **66**, 71-109 (2009).
12. Neese, F. Software update: The ORCA program system-version 5.0. *Wiley Interdiscip. Rev. Comput. Mol. Sci*. **12**, e1606 (2022).

13. Marcus, R. A. Electron transfer reactions in chemistry. Theory and experiment. *Rev. Mod. Phys*. **65**, 599-610 (1993).

14. Beljonne, D., Shuai, Z., Pourtois, G. & Bredas, J. L. Spin-orbit coupling and intersystem crossing in conjugated polymers: a configuration interaction description. *J. Phys. Chem. A* **105**, 3899-3907 (2001).

15. Kohler, A. & Bassler, H. Triplet states in organic semi-conductors. *Mater. Sci. Eng., R* **66**, 71-109 (2009).
